# Rapid Freezing Enables Aminoglycosides To Eradicate Bacterial Persisters via Enhancing Mechanosensitive Channel MscL-Mediated Antibiotic Uptake

**DOI:** 10.1128/mBio.03239-19

**Published:** 2020-02-11

**Authors:** Yanna Zhao, Boyan Lv, Fengqi Sun, Jiafeng Liu, Yan Wang, Yuanyuan Gao, Feng Qi, Zengyi Chang, Xinmiao Fu

**Affiliations:** a Provincial University Key Laboratory of Cellular Stress Response and Metabolic Regulation, Key Laboratory of Optoelectronic Science and Technology for Medicine of Ministry of Education, College of Life Sciences, Fujian Normal University, Fuzhou City, Fujian Province, China; b State Key Laboratory of Protein and Plant Gene Research, School of Life Sciences, Peking University, Beijing, China; c Engineering Research Center of Industrial Microbiology of Ministry of Education, Fujian Normal University, Fuzhou City, Fujian Province, China; Sequella, Inc.

**Keywords:** persister, antibiotic tolerance, aminoglycoside, freezing, antibiotic uptake, MscL, mechanosensitive ion channel, cryotherapy, *Pseudomonas aeruginosa*, antibiotic resistance, drug uptake, membrane channel proteins, persistence

## Abstract

Bacterial persisters exhibit noninherited antibiotic tolerance and are linked to the recalcitrance of bacterial infections. It is very urgent but also challenging to develop antipersister strategies. Here, we report that 10-s freezing with liquid nitrogen dramatically enhances the bactericidal action of aminoglycoside antibiotics by 2 to 6 orders of magnitude against many Gram-negative pathogens, with weaker potentiation effects on Gram-positive bacteria. In particular, antibiotic-tolerant Escherichia coli and Pseudomonas aeruginosa persisters—which were prepared by treating exponential-phase cells with ampicillin, ofloxacin, the protonophore cyanide *m*-chlorophenyl hydrazone (CCCP), or bacteriostatic antibiotics—can be effectively killed. We demonstrated, as a proof of concept, that freezing potentiated the aminoglycosides' killing of P. aeruginosa persisters in a mouse acute skin wound model. Mechanistically, freezing dramatically increased the bacterial uptake of aminoglycosides regardless of the presence of CCCP, indicating that the effects are independent of the proton motive force (PMF). In line with these results, we found that the effects were linked to freezing-induced cell membrane damage and were attributable, at least partly, to the mechanosensitive ion channel MscL, which was able to directly mediate such freezing-enhanced aminoglycoside uptake. In view of these results, we propose that the freezing-induced aminoglycoside potentiation is achieved by freezing-induced cell membrane destabilization, which, in turn, activates the MscL channel, which is able to effectively take up aminoglycosides in a PMF-independent manner. Our work may pave the way for the development of antipersister strategies that utilize the same mechanism as freezing but do so without causing any injury to animal cells.

## Abstract (precis)

Antibiotics have long been used to successfully kill bacterial pathogens, but antibiotic resistance/tolerance usually has led to the failure of antibiotic therapy, and it has become a severe threat to human health. How to improve the efficacy of existing antibiotics is of importance for combating antibiotic-resistant/tolerant pathogens. Here, we report that 10-s rapid freezing with liquid nitrogen dramatically enhanced the bactericidal action of aminoglycoside antibiotics by 2 to 6 orders of magnitude against many bacterial pathogens *in vitro* and also in a mouse skin wound model. In particular, such combined treatment was able to effectively kill persister cells of Escherichia coli and Pseudomonas aeruginosa, which are *per se* tolerant of conventional treatment with bactericidal antibiotics for several hours. We also demonstrated that freezing-induced aminoglycoside potentiation was apparently linked to freezing-induced cell membrane damage that may have activated the mechanosensitive ion channel MscL, which, in turn, was able to effectively uptake aminoglycoside antibiotics in a proton motive force-independent manner. Our report sheds light on the development of a new strategy against bacterial pathogens by combining existing antibiotics with a conventional physical treatment or with MscL agonists.

## INTRODUCTION

Curing infectious diseases caused by bacterial pathogens with antibiotics has been considered the most important medical achievement in the 20th century. However, we have also learned that bacteria are able to counteract the action of antibiotics through multiple ingenious mechanisms, including drug inactivation, alteration of drug targets, bypassing of the inhibited steps, and decreased uptake and/or increased efflux of the drug (1–3). In addition, bacteria are able to survive antibiotic attack by existing as persisters, which are genetically identical to the normal antibiotic-sensitive cells but exhibit a transient and nonheritable antibiotic tolerance (4–6). Such persister formation is widely believed to contribute to recurrent infections, as well as to the development of antibiotic resistance in pathogens (5, 7–11).

Antibiotic resistance of bacteria leads to the failure of antibiotic therapy and gives rise to life-threatening infections and has become a severe threat to global public health and economic development (12). Discovery and development of newer antibiotics have played a dominant role in this war, but as past experience indicates, resistance of pathogens to new drugs often occurs soon (12, 13). In particular, the pace of discovering new antibiotics has slowed dramatically since the 1980s, such that there has been a gap of more than 30 years since the discovery of new types of antibiotics (14, 15). This reflects both scientific and financial barriers to the identification of new antibiotics, and therefore the need to develop other strategies to fight antibiotic resistance is very urgent.

One effective strategy is to use traditional antibiotics in a wiser manner (2, 12, 14, 16, 17). In this respect, certain metabolites (e.g., glucose, mannitol, and alanine) have been reported to dramatically enhance the action of aminoglycosides against antibiotic-tolerant/resistant pathogens both *in vitro* and *in vivo* through increasing aminoglycoside uptake in a proton motive force (PMF)-dependent manner (18–22). Furthermore, inhibitors of efflux pumps have been widely reported to enhance the bactericidal action of various types of antibiotics by suppressing their outflow from bacteria (23, 24). Notably, we recently reported that hypoionic shock (i.e., treatment with ion-free solutions) could markedly potentiate aminoglycosides against Escherichia coli stationary-phase persisters (25). The aminoglycoside tobramycin has also been shown to be potentiated in combination with approved iron chelators (26) or the β-lactam aztreonam (27) for killing cystic fibrosis-related Pseudomonas aeruginosa. Other promising strategies, such as the use of membrane-active macromolecules (28), arginine-induced pH alteration (29), and osmotic compounds (30), have been reported to potentiate the existing antibiotics.

Here, we report, for the first time, that rapid freezing can dramatically and specifically enhance the bactericidal action of aminoglycoside antibiotics against normal cells and also antibiotic-tolerant persisters, both *in vitro* and in a mouse acute skin wound model. Remarkably, the aminoglycoside uptake of bacteria is enhanced by freezing in a PMF-independent manner, which is in contrast to the widely reported metabolite-stimulated aminoglycoside potentiation (18–21). The precise molecular mechanisms underlying such unusual potentiation remain unclear at present; our data indicate that the potentiation is linked to freezing-induced cell membrane damage and the MscL ion channel. Our observations pave the way for the development of promising strategies for persister eradication.

## RESULTS

### Freezing dramatically enhances the bactericidal action of aminoglycosides against both stationary-phase and exponential-phase E. coli cells.

We previously reported that application of hypoionic shock for only 1 min was able to enhance the bactericidal efficacy of aminoglycoside antibiotics against E. coli stationary-phase cells by 4 to 5 orders of magnitude (25). We explored other physical strategies (e.g., UV exposure, sonication, microwave exposure, and freezing) for aminoglycoside potentiation. In those experiments, we found that freezing was able to significantly enhance the efficacy of aminoglycoside antibiotics (including tobramycin, streptomycin, gentamicin, and kanamycin) in killing E. coli cells, while other treatments were found to have severe side effects and/or little synergistic effect with aminoglycosides (data not shown).

**FIG 1:**
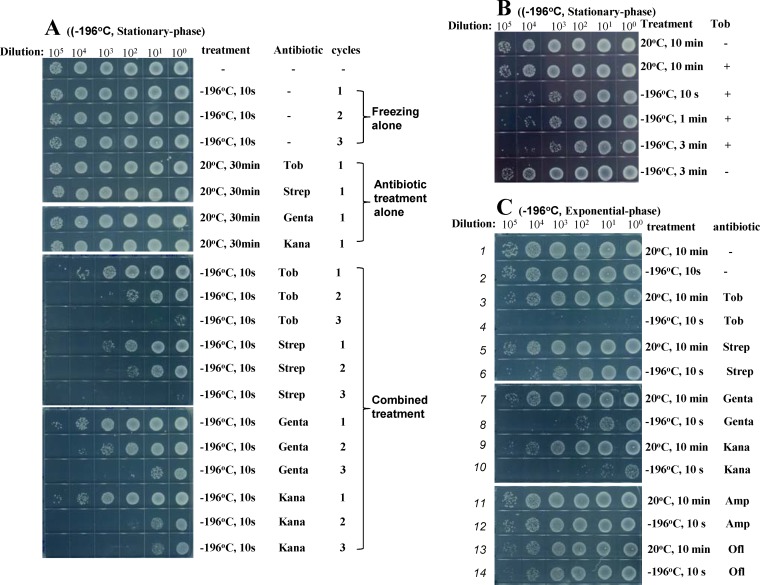
Freezing enhances the bactericidal action of aminoglycosides against both stationary-phase and exponential-phase E. coli cells. (A) Survival of E. coli stationary-phase cells following a 10-s treatment consisting of freezing in liquid nitrogen plus indicated aminoglycosides, with the treatment being cycled one, two, or three times. (B) Survival of E. coli stationary-phase cells following 10-s, 1-min, or 3-min treatment of freezing plus tobramycin. (C) Survival of E. coli exponential-phase cells following 10-s treatment of freezing plus indicated antibiotics. Tob, tobramycin; Strep, streptomycin; Genta, gentamicin; Kana, kanamycin; Amp, ampicillin; Ofl, ofloxacin. The treatment concentrations of the antibiotics are described in Table S1B.

First, we noticed that stationary-phase E. coli cells showed significantly reduced viability after the aminoglycoside-containing bacterial cultures were frozen in liquid nitrogen (−196°C; Fig. 1A) for 10 s or in ethanol prechilled at −80°C (see Fig. S1A in the supplemental material) for 20 s and subsequently thawed in an ice-water bath. When such combined treatments were repeated two or three times, cell viability was further reduced (Fig. 1A; see also Fig. S1A). Notably, such potentiation by freezing appears to be specific for aminoglycosides, without influencing two other classes of bactericidal antibiotics, i.e., β-lactams (ampicillin and carbenicillin) and fluoroquinolones (ofloxacin and ciprofloxacin) (Fig. S1B). Neither repeated freezing nor antibiotic treatment alone for 30 min at room temperature significantly killed the cells (Fig. 1A, top panel; see also Fig. S1A). In addition, extension of the duration of the combined treatment of tobramycin and freezing from 10 s to 3 min did not further reduce bacterial viability (Fig. 1B; see also Fig. S1C), implying that the molecular events accounting for such aminoglycoside potentiation take place during cooling and/or warming rather than during the frozen state.

10.1128/mBio.03239-19.1 FIG S1 Aminoglycoside potentiation by freezing against E. coli cells. Download FIG S1, PDF file, 0.2 MB. Copyright © 2020 Zhao et al. 2020 Zhao et al. This content is distributed under the terms of the Creative Commons Attribution 4.0 International license.

10.1128/mBio.03239-19.10 TABLE S1 (A) Bacterial strains used in this study. (B) Antibiotics and the concentrations used. Download Table S1, DOCX file, 0.01 MB. Copyright © 2020 Zhao et al. 2020 Zhao et al. This content is distributed under the terms of the Creative Commons Attribution 4.0 International license.

In further support of the aminoglycoside potentiation by freezing, stationary-phase E. coli cells largely survived after a 3-h treatment with aminoglycoside at 37°C (Fig. S1D, line 1 versus line 2 [except for streptomycin]); however, these surviving cells were effectively killed upon subsequent cycled freezing (Fig. S1D, line 2 versus lines 3 to 5). We also found that freezing performed only once, either at −196°C (Fig. 1C) or at −80°C (Fig. S1E), was able to dramatically enhance the bactericidal action of aminoglycosides against exponential-phase E. coli cells (lines 4, 6, 8, and 10 in Fig. 1C; see also Fig. S1E). Freezing alone (lines 2 in Fig. 1C; see also Fig. S1E) or antibiotic treatment alone for 10 min at 20°C (lines 3, 5, 7, and 9 in Fig. 1C; see also Fig. S1E) did not kill the bacterial cells at significant levels. Similarly, such potentiation by freezing is specific for aminoglycosides but not for β-lactams (ampicillin) or fluoroquinolones (ofloxacin) (lines 12 and 14 in Fig. 1C; see also Fig. S1E).

### Freezing enables aminoglycosides to eradicate antibiotic-tolerant E. coli persisters or persister-like cells in a PMF-independent manner.

**FIG 2:**
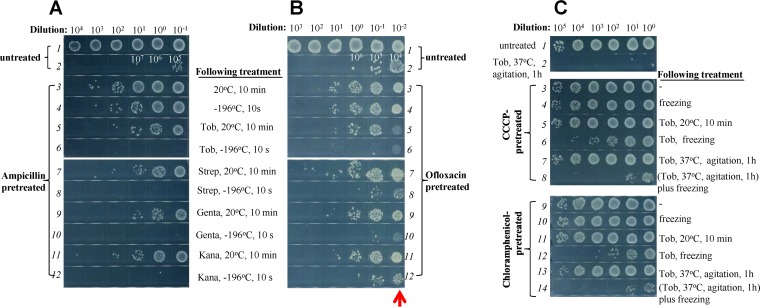
Freezing facilitates activity of aminoglycosides to effectively kill antibiotic-tolerant E. coli persisters or persister-like cells in a PMF-independent manner. (A and B) Survival of E. coli persister cells following a 10-s treatment consisting of freezing plus addition of the indicated aminoglycosides. Persister cells were prepared by pretreating exponential-phase cultures of E. coli with 100 μg/ml ampicillin (A) or 5 μg/ml ofloxacin (B) at 37°C for 3 h, and the remaining persister cells were concentrated 10-fold (i.e., a dilution of 10^−1^) (A) or 100-fold (i.e., a dilution of 10^−2^) (B) before being subjected to the combined treatment. Note that ofloxacin-treated cells, after 100-fold concentration, are extremely high in cell density and thus visible after spot plating (red arrow in panel B; see also Fig. S2A). (C) Survival of E. coli persister-like cells following 10-s treatment of freezing plus tobramycin. Persister-like cells were prepared by pretreating exponential-phase E. coli cells with 20 μM CCCP or 35 μg/ml chloramphenicol at 37°C for 1 h and then subjected to the combined treatment with tobramycin (25 μg/ml) and freezing (lines 6 and 12). Pretreated cells were also mixed with tobramycin and agitated at 37°C for 1 h (lines 7 and 13) before freezing (lines 8 and 14).

Bacterial persisters are widely believed to account for recurrent infections and antibiotic resistance development (5, 7–9, 31), and their eradication is of particular clinical interest. We prepared antibiotic-tolerant persister cells by treating exponential-phase cells with bactericidal antibiotics (represented by ampicillin and ofloxacin) according to earlier reports (32–34) and examined whether freezing can enhance the bactericidal effects of aminoglycosides on these persister cells. As expected, exponential-phase E. coli cells were mostly killed by ampicillin, with a small fraction (around 1/1,000) of original cells being ampicillin tolerant (line 3, Fig. 2A). These ampicillin-tolerant persister cells were fully eradicated by a combined treatment consisting of freezing and aminoglycoside (line 3 versus lines 6, 8, 10, and 12 in Fig. 2A) but showed only a marginal degree of killing by single treatments (line 3 versus lines 5, 7, 9, and 11). Similarly, a small fraction (around 1/1,000 to 1/10,000 of the total) of the E. coli exponential-phase cells were ofloxacin tolerant (line 3 in Fig. 2B), consistent with an earlier report (32), and these persisters were also effectively eradicated by the combined treatment (lines 6, 8, 10, and 12) but not by single treatments (lines 5, 7, 9, and 11).

10.1128/mBio.03239-19.2 FIG S2 Freezing facilitates aminoglycosides to eradicate antibiotic-tolerant E. coli persister-like cells independently of PMF. Download FIG S2, PDF file, 0.2 MB. Copyright © 2020 Zhao et al. 2020 Zhao et al. This content is distributed under the terms of the Creative Commons Attribution 4.0 International license.

Furthermore, we demonstrated that freezing also facilitates the killing by aminoglycosides of persister-like bacterial cells that were formed by chemical induction of antibiotic-sensitive cells. E. coli exponential-phase cells, initially highly sensitive to tobramycin (line 1 versus line 2 in Fig. 2C), become relatively tolerant to tobramycin (line 7) after pretreatment with the widely used protonophore carbonyl cyanide *m*-chlorophenyl hydrazone (CCCP) (35, 36). This reflects the fact that the aminoglycoside uptake by bacteria under conventional treatment conditions depends on the PMF, which can be dissipated by CCCP (18, 37, 38). Such persister-like cells, however, were effectively killed by a combined treatment consisting of addition of tobramycin and freezing (lines 6 and 8 in Fig. 2C) but not by single treatments (lines 3 and 4). We confirmed that CCCP pretreatment did decrease the PMF of E. coli cells as monitored by membrane potential probe-based flow cytometric analysis, and we observed that freezing itself partially impaired the PMF (Fig. S2B). Furthermore, pretreatment with FCCP (carbonyl cyanide-*p*-trifluoromethoxyphenylhydrazone; a functional analog of CCCP for PMF dissipation) was also able to suppress the bactericidal actions of tobramycin under conventional treatment conditions (line 7 versus line 2 in Fig. S2C) but did not suppress the freezing-induced tobramycin potentiation (lines 6 and 8). In view of these results, we conclude that the PMF is dispensable for the freezing-induced aminoglycoside potentiation. In further support of this notion, pretreatment with sodium azide, a widely used electron transport inhibitor, also enabled the exponential-phase E. coli cells to become tobramycin tolerant (line 7 versus line 2 in Fig. S2D), consistent with earlier studies (as reviewed in reference 38), and such persister-like cells were effectively killed by the combined treatment (lines 6 and 8) but not by single treatments (lines 4 and 5). We also confirmed that the intracellular ATP levels of E. coli exponential-phase cells were dramatically reduced after pretreatment with CCCP or sodium azide (Fig. S2E).

In addition, we observed that pretreatment of E. coli exponential-phase cells with bacteriostatic antibiotics such as chloramphenicol, erythromycin, and rifampin also increased the ratio of cells tolerant to tobramycin (line 13 in Fig. 2C; lines 5 and 11 in Fig. S2F), in line with earlier reports (35, 39). Again, such persister-like cells were effectively killed by the combined treatment consisting of tobramycin and freezing (lines 12 and 14 in Fig. 2C; lines 4, 6, 10, and 12 in Fig. S2F) but not by single treatments (lines 10 and 11 in Fig. 2C; lines 2, 3, 8, and 9 in Fig. S2F). Together, these observations demonstrate that freezing facilitates the killing by aminoglycoside antibiotics of not only antibiotic-sensitive cells but also antibiotic-tolerant persister and persister-like cells.

### Freezing dramatically enhances the aminoglycoside uptake of E. coli cells in a PMF-independent manner.

**FIG 3:**
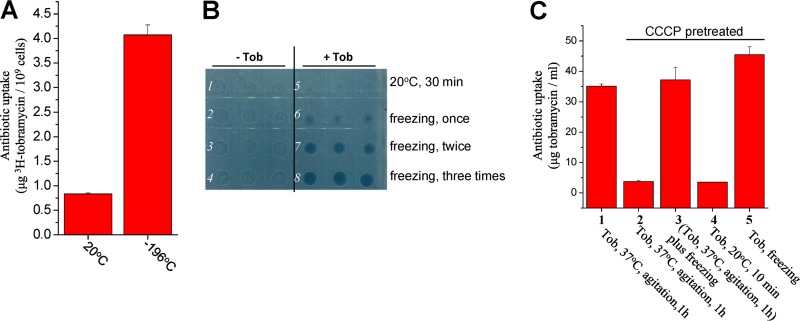
Freezing enhances the tobramycin uptake of E. coli cells in a PMF-independent manner. (A) Level of ^3^H-labeled tobramycin as taken up by E. coli exponential-phase cells following a 10-s treatment consisting of freezing in liquid nitrogen plus addition of ^3^H-labeled tobramycin (for details, see Materials and Methods). (B) Growth inhibition of E. coli cells on LB agar dishes by tobramycin, which was extracted from E. coli stationary-phase cells pretreated with tobramycin, and freezing in liquid nitrogen one, two, or three times. The procedure for tobramycin extraction was described in our earlier report (41). (C) Quantification of tobramycin taken up by CCCP-pretreated E. coli exponential-phase cells. Cells were treated as described for Fig. 2C and then subjected to tobramycin extraction. Quantification was performed based on the cell growth inhibition represented in Fig. S3C and the standard curve displayed in Fig. S3D. Data in panels A and C represent means ± SD of results from three replicates.

Prompted by earlier reports (18, 19, 22) revealing that metabolite-induced aminoglycoside potentiation is achieved by enhancing aminoglycoside uptake, we examined whether freezing could enhance the aminoglycoside uptake of E. coli cells via two independent methods. First, we applied a radioactivity assay as conventionally performed (38). Specifically, ^3^H-labeled tobramycin was mixed thoroughly with E. coli exponential-phase cells and then subjected to either freezing at −196°C for 10 s or continuous incubation at room temperature for 10 min. Results show that the freezing treatment enabled the cells to accumulate ^3^H-labeled tobramycin at a density of 4.07 ± 0.20 μg tobramycin/10^9^ cells, about 5-fold higher than that of cells incubated at room temperature (0.84 ± 0.02 μg tobramycin/10^9^ cells; Fig. 3A).

10.1128/mBio.03239-19.3 FIG S3 Freezing enhances the bacterial uptake of tobramycin. Download FIG S3, PDF file, 0.5 MB. Copyright © 2020 Zhao et al. 2020 Zhao et al. This content is distributed under the terms of the Creative Commons Attribution 4.0 International license.

Second, taking advantage of the high thermal stability of tobramycin (Fig. S3A) and the irreversible nature of aminoglycoside uptake by E. coli cells (40), we explored an alternative method for measuring tobramycin uptake as recently reported (41). Data presented in Fig. 3B indicate that tobramycin, as extracted from frozen E. coli stationary-phase cells, significantly inhibited bacterial growth (line 6 versus line 2), with the inhibition being stronger with more cycles of freezing (lines 6 to 8). In contrast, no significant inhibition of cell growth was observed for the tobramycin extracted from E. coli cells without the freezing treatment (line 5, Fig. 3B).

Furthermore, we demonstrated that freezing could enhance the tobramycin uptake of exponential-phase E. coli cells in a PMF-independent manner based on the following observations. First, the exponential-phase E. coli cells were able to effectively take up tobramycin after agitation at 37°C for 1 h (column 1 in Fig. 3C), in agreement with their high susceptibility to tobramycin (line 2 in Fig. 2C). Second, CCCP pretreatment, however, abolished this tobramycin uptake (column 2 in Fig. 3C), consistent with the fact that the PMF-dependent aminoglycoside uptake can be disrupted by CCCP (18, 37, 38). Third, when such CCCP-pretreated E. coli cells were agitated with tobramycin at 37°C for 1 h and then subjected to freezing, the tobramycin uptake was fully recovered (column 3 in Fig. 3C). Last, the tobramycin uptake was minimal when the CCCP-pretreated cells were incubated with tobramycin at room temperature for 10 min (column 4 in Fig. 3C) but was dramatically increased upon freezing (column 5 in Fig. 3C). These observations, together with the results indicating that freezing itself partially impaired the PMF (Fig. S2B), indicate that the PMF is dispensable for the freezing-enhanced uptake of tobramycin.

In addition, we also observed that pretreatment with chloramphenicol significantly decreased the tobramycin uptake of exponential-phase E. coli cells under conventional treatment conditions (column 2 versus column 1 in Fig. S3E). Nevertheless, an additional freezing treatment was able to fully recover the capacity of the cells to take up tobramycin (column 3 in Fig. S3E). Direct freezing of the chloramphenicol-pretreated cells also dramatically increased the tobramycin uptake (column 5 versus column 4 in Fig. S3E). These results agree well with the aforementioned cell survival assay results (Fig. 2C), indicating that protein synthesis is not a prerequisite for the freezing-enhanced aminoglycoside uptake, a scenario opposite that seen in the conventional aminoglycoside treatment (38).

### Freezing facilitates killing of many strains of Gram-negative bacteria by aminoglycosides, with weaker effects on Gram-positive bacteria.

**FIG 4:**
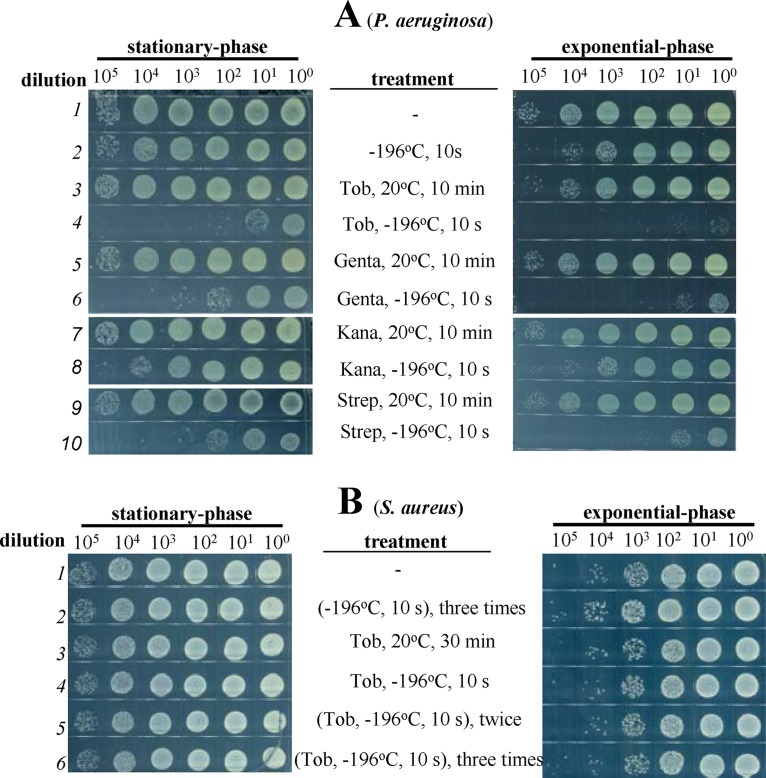
Freezing facilitates aminoglycoside killing of P. aeruginosa cells but not S. aureus cells. (A and B) Survival of stationary-phase (left) and exponential-phase (right) P. aeruginosa cells (A) and S. aureus cells (B) following a 10-s treatment consisting of freezing in liquid nitrogen plus addition of the indicated antibiotics at the concentrations described in Table S1B. Stationary-phase P. aeruginosa was frozen only once.

To explore the clinical potential of the combined treatments of aminoglycosides and freezing, we examined whether typical bacterial pathogens, such as the Gram-negative bacterium P. aeruginosa and the Gram-positive bacterium Staphylococcus aureus, could be killed. Results (Fig. 4A) indicate that both stationary-phase and exponential-phase P. aeruginosa cells are highly sensitive to the combined treatments (lines 4, 6, and 10) but not to tobramycin, gentamicin, or streptomycin treatment alone at room temperature (lines 3, 6, and 9). Again, freezing was unable to enhance the bactericidal action of β-lactams (ampicillin and carbenicillin) and fluoroquinolone (ofloxacin and ciprofloxacin) against P. aeruginosa cells (Fig. S4A). Freezing enhanced the action of kanamycin only minimally (line 8, Fig. 4A), presumably due to the intrinsic resistance of P. aeruginosa cells to kanamycin (left part in Fig. S4B). In contrast to P. aeruginosa, neither stationary-phase nor exponential-phase S. aureus cells were found to be sensitive to the combined treatment consisting of aminoglycoside (tobramycin) and freezing (lines 4, 5, and 6 in Fig. 4B), although exponential-phase S. aureus cells *per se* were sensitive to tobramycin under conventional treatment conditions (Fig. S4B).

10.1128/mBio.03239-19.4 FIG S4 Antibiotic sensitivity of various bacterial strains. Download FIG S4, PDF file, 0.5 MB. Copyright © 2020 Zhao et al. 2020 Zhao et al. This content is distributed under the terms of the Creative Commons Attribution 4.0 International license.

We then expanded our test to many other Gram-negative and Gram-positive bacteria in both exponential-phase and stationary-phase states. The levels of sensitivity of these bacteria to the four types of aminoglycoside antibiotics are shown in Fig. S4C. Results of analysis of five more Gram-negative bacteria (Fig. S5A) indicate that (i) Acinetobacter baumannii Ab6 and Klebsiella pneumoniae KP-D367 are insensitive, presumably because they are resistant to these aminoglycoside antibiotics *per se* (Fig. S4C), and (ii) Shigella flexneri 24T7T and Salmonella enterica serovar Typhimurium SL1344 are sensitive to the combined treatments consisting of certain aminoglycosides and freezing in their exponential-phase and/or stationary-phase states.

10.1128/mBio.03239-19.5 FIG S5 Freezing potentiates aminoglycosides against exponential- and/or stationary-phase cells of several bacterial strains. Download FIG S5, PDF file, 0.3 MB. Copyright © 2020 Zhao et al. 2020 Zhao et al. This content is distributed under the terms of the Creative Commons Attribution 4.0 International license.

Results of analysis of five more Gram-positive bacterial species (Fig. S5B) indicate that (i) Lactococcus lactis NZ9000 and Enterococcus faecalis ATCC 29212 are insensitive, presumably because they are resistant to these aminoglycoside antibiotics *per se* (Fig. S4C); (ii) Bacillus subtilis is sensitive in its exponential-phase stage but not in its stationary-phase stage; (iii) Staphylococcus epidermidis CMCC26069 is only slightly sensitive in its stationary-phase stage; and (iv) Micrococcus luteus CMCC 28001 is insensitive. The levels of sensitivity of each bacterium to antibiotic alone and to combined treatment are summarized in Table 1. These studies, though not exhaustive, suggested that, overall, Gram-negative bacteria are more sensitive to the combined treatments of aminoglycoside and freezing than Gram-positive bacteria.

**TABLE 1:** Sensitivity of various bacterial strains to combined aminoglycoside and freezing treatment

Bacterial species	Sensitivity to particular antibiotics^*a*^							
	Tom		Genta		Strep		Kana	
	A	C	A	C	A	C	A	C
Escherichia coli	++	+++/+++	++	++/++	++	++/+++	++	++/++
Pseudomonas aeruginosa	++	++/++	++	++/++	++	++/++	−	−/−
Acinetobacter baumannii	−	−/−	−	−/−	−	−/−	−	−/−
Klebsiella pneumoniae	−	−/−	−	−/−	−	−/−	−	−/−
Salmonella enterica serovar Typhimurium	++	++/−	++	++/−	−	−/−	++	++/−
Shigella flexneri	++	+/++	++	−/++	−	−/−	++	+/++
Staphylococcus aureus	++	−/−	+	−/−	−	−/−	+	−/−
Lactococcus lactis	−	−/−	−	−/−	−	−/−	−	−/−
Enterococcus faecalis	−	−/−	−	−/−	−/	−/−	−	−/−
Bacillus subtilis	++	+/−	++	+/−	++	+/−	++	+/−
Staphylococcus epidermidis	++	−/+	+	−/−	−	−/+	+	−/+
Micrococcus luteus	−	−/−	++	−/−	++	−/−	−	−/−

a A, antibiotic sensitivity of each bacterium under conditions of agitation of cells in the exponential-phase state at 37°C for 1 h (for P. aeruginosa and S. aureus; refer to Fig. S4B) or for 2 h (for other strains; refer to Fig. S4C) in the presence of the antibiotics. C, combined treatment. For each bacterium, both the exponential- and the stationary-phase cells were subjected to a combined treatment of antibiotic plus freezing (–196°C for 10 s), with the exponential-phase cells being frozen once and the stationary-phase cells (with the exception of P. aeruginosa; Fig. 4A) three times, and the cells were then subjected to the bacterial survival assay (as presented in Fig. S5). The levels of sensitivity of the exponential-phase cells and the stationary-phase cells are indicated before and after the slash (“/”), respectively, and are defined as follows: +++, highly sensitive; ++, sensitive; +, slightly sensitive; −, insensitive. Tom, tobramycin; Genta, gentamicin; Strep, streptomycin; Kana, kanamycin.

### Freezing facilitates killing of P. aeruginosa by aminoglycoside persisters in mice.

P. aeruginosa is a multidrug-resistant pathogen associated with serious illnesses such as cystic fibrosis and traumatic burns. It was reported previously that isolates of P. aeruginosa from patients at a late stage of cystic fibrosis produce higher levels of antibiotic-tolerant persister cells and that such increased persister formation is their sole mechanism for surviving chemotherapy (5, 42). Here, we prepared P. aeruginosa persisters by the use of the same method as that used on the E. coli cells and found that the combined treatment of aminoglycoside and freezing was able to eradicate such P. aeruginosa persisters in a PMF-independent manner (Fig. S6), a scenario similar to the aforementioned observations on E. coli persisters (Fig. 2; see also Fig. S2).

10.1128/mBio.03239-19.6 FIG S6 Freezing facilitates aminoglycosides to kill P. aeruginosa persisters independently of PMF. Download FIG S6, PDF file, 0.5 MB. Copyright © 2020 Zhao et al. 2020 Zhao et al. This content is distributed under the terms of the Creative Commons Attribution 4.0 International license.

**FIG 5:**
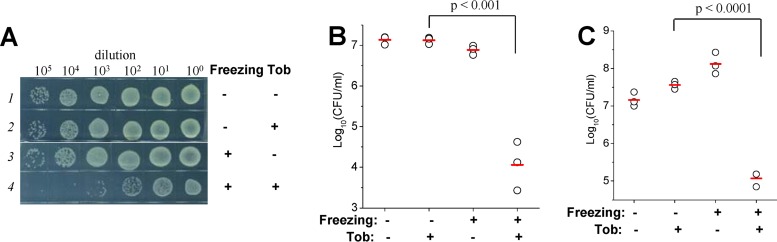
Freezing facilitates tobramycin killing of P. aeruginosa in a mouse model. (A) Survival of stationary-phase P. aeruginosa cells after the cells were mixed with 100 μg/ml tobramycin, plated on a piece of mouse muscle, and subjected to freezing in liquid nitrogen for 1 min. Cells were taken from the muscle and plated onto LB agar dishes. (B) Survival of stationary-phase P. aeruginosa cells after the cells were mixed with 100 μg/ml tobramycin, plated on the tail of an anesthetized mouse, and subjected to freezing in liquid nitrogen for 2 min. Tail lysates were plated on LB agar dishes for cell survival assay. (C) Survival of CCCP-pretreated P. aeruginosa cells in the wounds of mice. Cell survival assay results are presented in the middle part of Fig. S7E. Data in panels B and C are presented as means ± SD of results from three replicates.

Freezing (cryotherapy or cryosurgery) has been widely and long applied as a physical therapy to treat a number of diseases and disorders (43, 44) and even to treat skin, prostate, and lung cancers (43–46), although it damages animal cells and tissues (47, 48). Here, we demonstrated, as a proof of concept, that freezing was able to facilitate aminoglycoside eradication of P. aeruginosa persisters in animals based on the following observations. First, we removed muscles from sacrificed mice and then placed stationary-phase P. aeruginosa cells (premixed with tobramycin) on the muscles before freezing them with liquid nitrogen. Our bacterial survival assay showed that freezing significantly potentiated tobramycin against P. aeruginosa cells under such *in situ* conditions (column 4 versus column 3 in Fig. 5A).

10.1128/mBio.03239-19.7 FIG S7 Freezing facilitates tobramycin to kill P. aeruginosa in mouse model. Download FIG S7, PDF file, 0.5 MB. Copyright © 2020 Zhao et al. 2020 Zhao et al. This content is distributed under the terms of the Creative Commons Attribution 4.0 International license.

Second, we employed the mouse tail as a model, considering that it is convenient to immerse the tail of a live animal in liquid nitrogen. To this end, we anesthetized mice, placed stationary-phase P. aeruginosa cell culture containing tobramycin on the tails, wrapped the tails with Parafilm, and immersed them in liquid nitrogen (refer to Fig. S7A). Bacterial survival assay results show that freezing at −196°C significantly (*P < *0.001) enhanced the bactericidal effects of tobramycin on P. aeruginosa cells in comparison with room temperature (Fig. 5B [see also Fig. S7B]).

Third, we utilized an acute skin wound model (49) to show the *in vivo* efficacy of the combined treatment. To this end, a piece of skin 1 cm by 1 cm in size was removed from the right back of anesthetized mice. P. aeruginosa cells were placed on the wound, tobramycin-containing culture medium was added, and the wound was subsequently subjected to a freezing treatment and bandaged (Fig. S7C). Initially, we examined stationary-phase P. aeruginosa cells and observed only a marginal freezing-induced tobramycin potentiation effect (Fig. S7D). Then we examined exponential-phase P. aeruginosa cells and observed a substantial tobramycin potentiation effect of freezing (Fig. S7E, left), particularly against both CCCP-pretreated and chloramphenicol-pretreated cells (middle and right parts). Quantification analysis revealed that freezing could significantly (*P < *0.0001) potentiate tobramycin against CCCP-pretreated P. aeruginosa cells in an acute skin wound model (Fig. 5C). As expected, the frozen wounds became black overnight postsurgery (Fig. S7F), presumably due to freezing injury.

### Freezing-induced aminoglycoside potentiation is suppressed by decreasing the cooling rate, by increasing the warming rate, and by the presence of cryoprotectants.

We attempted to uncover the molecular mechanism underlying freezing-induced aminoglycoside potentiation. Nevertheless, these efforts were substantially compromised by our current limited understanding of the mechanism of freezing injury on cells (47, 48, 50–52). The extent of the effect of freezing injury on specific cells seems to depend on three major variables: the cooling rate, the warming rate, and the type and concentration of protective additives (48). Therefore, we examined the effects of these variables.

**FIG 6:**
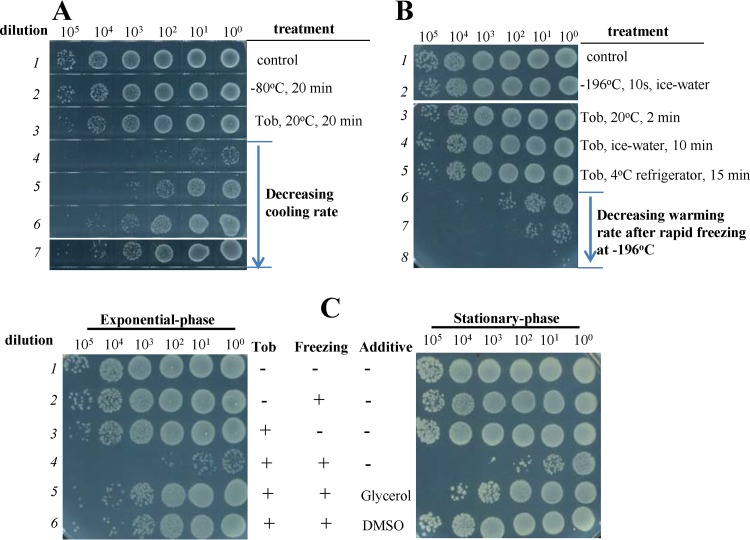
Effects of cooling rate, warming rate, and cryoprotectants on tobramycin potentiation. (A) Survival of exponential-phase E. coli cells following freezing treatment in ethanol prechilled at −80°C (line 4) or in a −80°C freezer without (line 5) or with (lines 6 and 7) an additional package of tubes (for details, see Materials and Methods), in the presence of 25 μg/ml tobramycin. (B) Survival of exponential-phase E. coli cells after the cells were frozen in liquid nitrogen for 10 s in the presence of 25 μg/ml tobramycin and then thawed at different warming rates (line 6, a 30°C water bath; line 7, an ice-water bath; line 8, a 4°C refrigerator). (C) Survival of exponential-phase (left) and stationary-phase (right) E. coli cells after the cells were mixed with tobramycin at concentrations of 25 and 100 μg/ml, respectively, and then subjected to freezing/thawing treatment once or three times, respectively, in the presence of 20% glycerol or 10% DMSO.

First, we show that the cooling rate is critical for the freezing-induced aminoglycoside potentiation. When the cooling rate of freezing was decreased by placing the bacterium-containing PCR tube in a −80°C freezer rather than immersing it in ethanol at −80°C, the bactericidal effects of tobramycin were remarkably compromised (line 5 versus line 4 in Fig. 6A). An additional decrease in the cooling rate resulted in a much lower level of cell death (lines 6 and 7 in Fig. 6A). These observations indicate that rapid freezing potentiates aminoglycoside much more effectively than slow freezing, illustrating a critical role of intracellular ice formation in bacteria in the potentiation effect according to cryobiological studies (48, 53).

Second, we found that the freezing-induced aminoglycoside potentiation was augmented if the rapidly frozen cells were thawed slowly. When the rapidly frozen E. coli cells were thawed in a 30°C water bath (line 6 in Fig. 6B), in an ice-water bath (line 7), or in a 4°C refrigerator (line 8), the time required for their full thawing was around 1.5, 10, or 15 min, respectively, and the cell death ratio was significantly augmented upon slower warming. This observation, in conjunction with the general views from cryobiological studies (48, 52), suggests that recrystallization of intracellular ice crystals due to slow warming plays a critical role in freezing-induced aminoglycoside potentiation.

According to cryobiological studies (50, 52), the freezing behavior of cells can be modified by cryoprotectants, which affect the rates of water transport, nucleation, and crystal growth and collectively reduce freezing injury and cell death. Here, we found that glycerol and dimethyl sulfoxide (DMSO), two cryoprotectants widely used for cell preservation (53), largely diminished the freezing-induced aminoglycoside potentiation for both exponential-phase and stationary-phase E. coli cells (lines 6 and 5 versus line 4 in Fig. 6C). Collectively, these observations suggest that the freezing-induced aminoglycoside potentiation is linked to the freezing injury to bacterial cells.

### Freezing-induced aminoglycoside potentiation is linked to freezing injury of the cell membrane.

**FIG 7:**
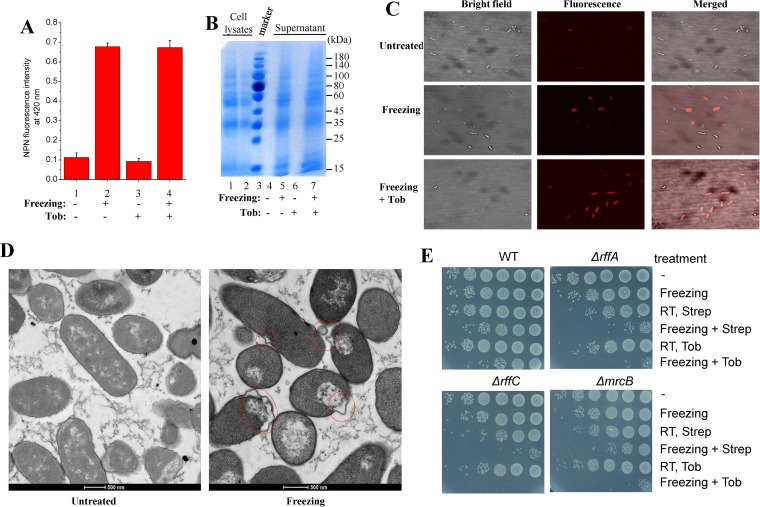
E. coli cell membranes are damaged upon freezing. (A) Fluorescence intensity of NPN after a 5-min incubation with stationary-phase E. coli cells. Cells were resuspended in HEPES buffer and subjected to three cycles of freezing in liquid nitrogen and thawing in ice water before mixing with 10 μM NPN was performed. (B) SDS-PAGE analysis results from the supernatant of stationary-phase E. coli cells after undergoing indicated treatments. Proteins in the supernatant were concentrated 10-fold by precipitation with 10% trichloroacetic acid before electrophoresis. Lane 1, whole-cell extract; lane 2, 2-fold diluted whole-cell extracts. (C) Fluorescence microscopy imaging of exponential-phase E. coli cells, which were frozen, thawed, and incubated with 50 μg/ml PI for 10 min before imaging. (D) Transmission electron micrographs of exponential-phase E. coli cells following freezing treatment. (E) Survival of exponential-phase E. coli cells of the indicated genotypes following 10-s treatment with tobramycin or streptomycin plus freezing in liquid nitrogen. Results for other mutant strains are presented in Fig. S8C. RT, room temperature; WT, wild type.

Prompted by cryobiological studies revealing that the cell membrane (mostly in eukaryotic cells) is the primary site of freezing injury (48, 50, 53, 54), we examined whether freezing causes damage to E. coli cell membranes by a combination of membrane integrity staining, protein leakage assay, membrane permeability assay, and membrane morphology analysis. To this end, first, we found that the hydrophobic fluorescent probe 1-N-phenylnaphthylamine (NPN), which fluoresces weakly in aqueous environments but becomes strongly fluorescent when interacting with the hydrophobic tail of membrane phospholipids (55), exhibited a much higher fluorescence intensity upon binding to the frozen E. coli cells than to untreated cells (column 2 versus column 1 and column 4 versus column 3 in Fig. 7A). This shows that the cell membrane of the frozen E. coli cells was damaged to a certain extent.

10.1128/mBio.03239-19.8 FIG S8 Freezing-induced E. coli cell membrane damage and implications for the involvement of *mscL* gene in freezing-induced aminoglycoside potentiation. Download FIG S8, PDF file, 0.6 MB. Copyright © 2020 Zhao et al. 2020 Zhao et al. This content is distributed under the terms of the Creative Commons Attribution 4.0 International license.

Second, we examined the effects of freezing on the leakage of intracellular proteins. Results of SDS-PAGE analysis of proteins present in the cell suspension medium indicate that freezing treatment enables a significant portion (around 10%) of cellular proteins to leak out (lanes 5 and 7 in Fig. 7B), in line with earlier reports showing that intracellular content of bacterial, mammalian, and plant cells had leaked out after freezing (56–59). In contrast, unfrozen cells, in either the absence or presence of tobramycin, did not show any detectable cellular proteins in the medium (lanes 4 and 6 in Fig. 7B).

Third, we evaluated the cell membrane permeability of E. coli cells upon freezing by using propidium iodide (PI), a fluorescent probe widely used for DNA staining in membrane-permeabilized cells (60) (particularly dead cells). We found that many more E. coli cells were stained by PI after freezing than in the absence of freezing treatment, as revealed by fluorescence microscopic analysis (Fig. 7C) and flow cytometry analysis (Fig. S8A). Last but not least, thin-section transmission electron microscopic analysis revealed that the untreated E. coli cells exhibited regular cell morphology, while the frozen cells showed abnormalities in their cell envelopes, including the appearance of vesicles, bulges, and wrinkles (as highlighted by red circles in Fig. 7D).

Furthermore, we provide genetic evidence to show that aminoglycoside potentiation is linked to freezing injury on the cell membrane of bacteria. Initially, the Keio collection (a widely used E. coli genome-wide single-gene deletion library [61]) was subjected to freezing sensitivity screening test in our laboratory (data to be published elsewhere). Forty-one mutants were identified as hypersensitive, with deletion of genes related to ribosome biogenesis, tRNA modification, cell division, and membrane biogenesis and stability (Fig. S8B). Of these 41 mutants, some mutants potentially have a destabilized cell membrane due to a defect in lipopolysaccharide biosynthesis (*ΔrffA*, *ΔrffC*, *ΔrffD*, and *Δrfe* mutants), peptidoglycan biosynthesis (*ΔmrcB* mutant), or membrane protein biogenesis (*ΔdegP* mutant); these were selected for further examination. The *ΔrffA*, *ΔrffC*, and *ΔmrcB* mutants were much more sensitive to the combined treatment of aminoglycoside (streptomycin and/or tobramycin) and freezing than the BW25113 wild-type strain (Fig. 7E), and the *ΔrffD*, *Δrfe*, and *ΔdegP* mutants were also more sensitive (Fig. S8C). Collectively, these observations demonstrate that the cell membrane of E. coli cells is substantially damaged upon freezing and thawing, which may directly or indirectly contribute to the freezing-enhanced aminoglycoside uptake and thus to potentiation.

### The mechanosensitive ion channel MscL directly mediates the freezing-enhanced bacterial uptake of aminoglycoside.

Previously, Blount and coworkers uncovered a direct role of the mechanosensitive ion channel MscL in transportation of the aminoglycoside dihydrostreptomycin under conventional treatment conditions (62, 63). We thus systematically examined E. coli mutants lacking the *mscL* gene or other *mscL*-related, *mscS*-like mechanosensitive ion channel genes (i.e., *mscS*, *mscK*, *mscM*, *ynaI*, *ybdG*, and *ybiO*) (64). Nevertheless, none of these single-gene deletion mutants exhibited increased resistance to the combined treatment of aminoglycoside and freezing at the stationary-growth state (Fig. S8D).

**FIG 8:**
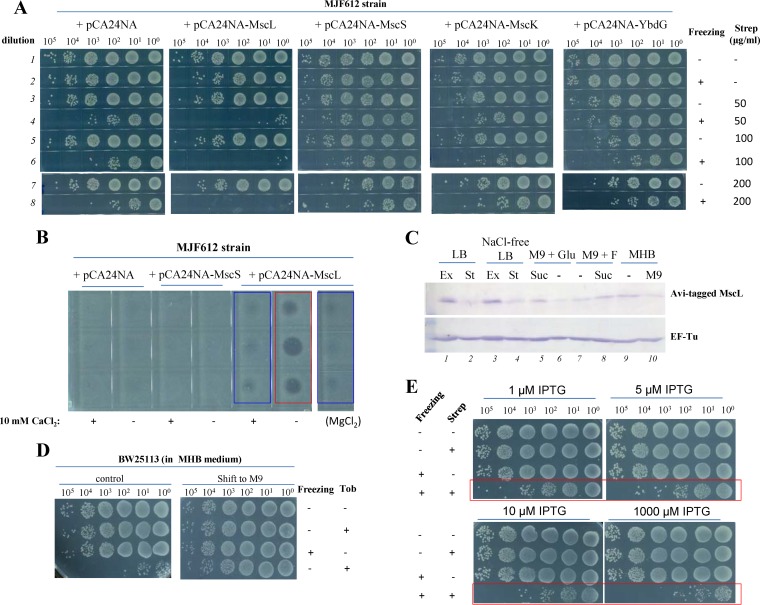
The MscL channel mediates the uptake of streptomycin by E. coli cells upon freezing. (A) Survival of exponential-phase E. coli cells of the indicated genotypes. E. coli MJF612 cells (Frag1 *ΔmscL*::cm, *ΔmscS*, *ΔmscK*::kan, *ΔybdG*::*aprD*) were transformed with the indicated plasmids for expressing each channel and then subjected to the combined treatment consisting of addition of streptomycin and freezing before cell survival assay. (B) Growth inhibition of E. coli cells on LB agar dishes by streptomycin as extracted from E. coli cells of the indicated genotypes that had been treated as indicated. (C) Immunoblotting analysis results showing the protein levels of endogenously expressed MscL in E. coli cells under the indicated culturing conditions. EF-Tu was analyzed as a sample-loading control. Ex, exponential-phase growth; St, stationary-phase growth; Suc, osmotic shock with 1.25 M sucrose; Glu, 5 g/liter glucose; F, 2 g/liter fumarate; “-”, untreated. (D) Survival of stationary-phase E. coli BW25113 cells, which were cultured in MHB medium, transferred to M9 medium, and cultured for 5 h before being subjected to the combined treatment of tobramycin and freezing. (E) Survival of MscL-expressing E. coli MJF612 cells following the combined treatment consisting of addition of streptomycin and freezing. MscL expression was induced to different degrees (Fig. S9D) by the indicated concentrations of IPTG.

We did observe, however, a marginal increase in the resistance to the combined treatment for *△mscL* exponential-phase cells (Fig. S8E). Prompted by this observation, we analyzed the effects of MscL on E. coli MJF612, which lacks the genes of four ion channels (i.e., *mscL*, *mscS*, *mscK*, and *ybdG*) (62, 65). Data presented in Fig. 8A indicate that complementary expression of MscL, but not of MscS, MscK, or YbdG, dramatically increased the sensitivity of MJF612 to the combined treatment consisting of addition of streptomycin and freezing (only streptomycin was analyzed due to the cross-resistance of MJF612 to tobramycin and gentamicin as a result of the presence of its kanamycin resistance gene; Fig. S8F). Consistently, we found that streptomycin, as extracted from frozen MJF612 cells complementarily expressing MscL but not MscS, suppressed bacterial cell growth (red frame in Fig. 8B), thus indicating a direct role of MscL in transporting streptomycin during freezing treatment.

10.1128/mBio.03239-19.9 FIG S9 MscL channel mediates the uptake of streptomycin in E. coli cells upon freezing and such uptake is inhibited by Ca^2+^/Mg^2+^. Download FIG S9, PDF file, 0.3 MB. Copyright © 2020 Zhao et al. 2020 Zhao et al. This content is distributed under the terms of the Creative Commons Attribution 4.0 International license.

To further demonstrate that MscL directly mediates aminoglycoside uptake of E. coli cells upon freezing and also identify the physiological conditions under which MscL significantly contributes to the freezing-induced aminoglycoside potentiation, we screened E. coli wild-type cells (BW25113 strain) in different states and examined whether their sensitivity to the combined treatment of tobramycin and freezing was correlated with the protein levels of endogenously expressed MscL. First, we found that E. coli exponential-phase cells, which were cultured either in normal LB medium or in NaCl-free LB medium, were much more sensitive to the combined treatment than stationary-phase cells (Fig. S9A). Meanwhile, the MscL protein levels in the exponential-phase cells were higher than in stationary-phase cells (lane 1 versus lane 2 and lane 3 versus lane 4 in Fig. 8C).

Second, when stationary-phase E. coli cells cultured in Mueller-Hinton broth (MHB) medium were transferred to M9 medium for preparation of starvation-induced persisters (41), they became much more tolerant to the combined treatment (Fig. 8D), and the MscL protein levels concomitantly decreased (lane 9 versus lane 10 in Fig. 8C). Third, E. coli exponential-phase cells cultured in M9 medium plus glucose became much more sensitive to the combined treatment after sucrose-mediated hyperosmotic shock (Fig. S9B), and the MscL protein levels concomitantly increased (lane 6 versus lane 5 in Fig. 8C). The same was observed for nutrient shift-induced E. coli persisters (Fig. S9C; lane 7 versus lane 8 in Fig. 8C), which were prepared by transferring E. coli exponential-phase cells from M9 medium plus glucose to M9 medium plus fumarate medium (41).

Last, we artificially induced MscL protein expression in E. coli MJF612 cells with increasing concentrations (1 μM, 5 μM, 10 μM, and 1,000 μM) of IPTG (isopropyl β-d-1-thiogalactopyranoside). With increasing IPTG concentrations, the MscL protein levels increased (lanes 1 to 4 in Fig. S9D), and the cells were increasingly sensitive to the combined treatment consisting of addition of streptomycin and freezing (red frame in Fig. 8E). These data, together with the results reported previously by Blount and coworkers showing a role of MscL in transporting streptomycin under conventional treatment conditions (62, 63), strongly suggest that MscL directly mediates the uptake and potentiation of aminoglycosides during freezing treatment.

### MscL-mediated aminoglycoside uptake upon freezing is inhibited by Ca^2+^/Mg^2+^.

**FIG 9:**
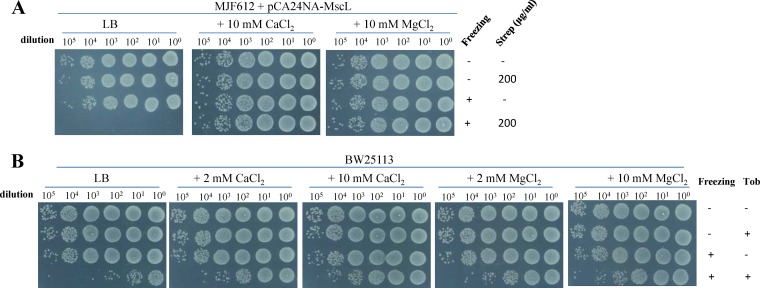
MscL-mediated uptake of streptomycin upon freezing is suppressed by Ca^2+^/Mg^2+^. (A and B) Survival of exponential-phase E. coli MJF612 cells complementarily expressing MscL (A) and BW25113 cells (B) following a 10-s treatment consisting of addition of streptomycin and freezing in the absence or presence of CaCl_2_ or MgCl_2_.

In the streptomycin uptake assay, we observed that the presence of 10 mM Ca^2+^ or Mg^2+^ significantly reduced the MscL-mediated uptake of streptomycin in E. coli MJF612 cells (blue frames versus red frame in Fig. 8B). In line with these observations, the presence of 10 mM Ca^2+^ or Mg^2+^ also abolished the freezing-induced streptomycin potentiation in MJF612 cells complementarily expressing MscL (Fig. 9A). These observations thus provide additional evidence that MscL mediates aminoglycoside uptake under freezing conditions. To evaluate the effects of Ca^2+^ and Mg^2+^ in the context of a wild-type genetic background, we analyzed E. coli BW25113 cells. Our results (Fig. 9B) indicate that both Ca^2+^ and Mg^2+^ were able to significantly suppress the freezing-induced aminoglycoside potentiation (Fig. 9A), while K^+^ and Na^+^ had no significant effects (Fig. S9E).

## DISCUSSION

The major findings in our study include (i) the potentiation effect of rapid freezing on aminoglycoside action against both normal bacterial cells and antibiotic-tolerant persisters (Fig. 1, 2, and 4; see also Fig. S6 in the supplemental material) (Table 1) and (ii) a direct role of the mechanosensitive ion channel MscL in mediating such potentiation (Fig. 8 and 9; see also Fig. S9). Not surprisingly, the potentiation effect is achieved by enhancing the bacterial uptake of aminoglycosides. Remarkably, this freezing-enhanced uptake is PMF and ATP independent (Fig. 2C and 3C; see also Fig. S2 and S6). These results contrast with the metabolite-stimulated PMF-dependent potentiation of aminoglycosides (18–22) and the PMF-dependent bacterial uptake of aminoglycosides under conventional conditions (as extensively reviewed in reference 38).

### Cell membrane destabilization-induced MscL activation contributes to freezing-enhanced aminoglycoside uptake.

Although aminoglycoside antibiotics have been used clinically since the antituberculosis drug streptomycin was discovered in the 1940s, their mechanisms of action remain enigmatic (18, 66–68), including how they are taken up by bacterial cells (38). It is generally accepted that the bacterial uptake of aminoglycosides is initiated by their ionic binding to the surface of bacterial cells, followed by two energy-dependent phases, i.e., EPDI and EPDII, which represent a low rate of energized uptake and a rapid energy-dependent accumulation of aminoglycosides that also requires protein synthesis, respectively (38). Nevertheless, the nature of transporters or carriers responsible for such energy-dependent translocation of the positively charged aminoglycosides across the cytoplasmic membrane remains largely undefined (37, 38), and elucidation of the form of the energy driving the bacterial uptake of aminoglycosides also remains elusive. Although the PMF has been known to be a requisite for the uptake (reference 38 and references therein), the issue of whether ATP and electron transfer play a role in driving aminoglycoside uptake has not been fully resolved (69).

Our combined treatments with aminoglycoside and freezing differ from earlier reports in the following aspects. (i) The aminoglycoside exposure time in our treatment was shortened to a few minutes, rather than the couple of hours required for metabolite-induced aminoglycoside potentiation (18–21) or in other more routine methods (32, 34). (ii) The bactericidal effect and bacterial uptake of aminoglycosides in our study were unaffected by the presence of CCCP or FCCP, i.e., these effects were PMF independent. (iii) Unlike EPDII, which requires protein synthesis (as reviewed in reference 38), the enhanced aminoglycoside uptake in our protocol does not require protein synthesis (Fig. 2C; see also Fig. S2F and S6E). These revelations strongly suggest that our combined treatment approach represents a totally different scenario.

The molecular mechanisms underlying this aminoglycoside uptake are apparently linked to intracellular ice formation-induced cellular injury occurring during freezing and also during thawing, in view of the general concepts in cryobiology (48, 52, 53, 70, 71) and our observations that freezing-induced aminoglycoside potentiation was suppressed by decreasing the cooling rate (Fig. 6A), increasing the warming rate (Fig. 6B), or adding cryoprotectants (Fig. 6C). On the other hand, cell membranes (including the cytoplasmic membrane and organelle membranes in eukaryotic cells) are generally thought to be the primary site of freezing injury according to cryobiological studies (48, 50, 53, 54, 71). In line with this notion, our combined analyses by NPN staining, PI staining, protein leakage assay, and electron microscopy collectively and unambiguously revealed the injurious effects of freezing on the membranes of E. coli cells (Fig. 7). In particular, some membrane-destabilized E. coli mutants are more sensitive to freezing-induced aminoglycoside killing (Fig. 7E; see also Fig. S8C). In light of all these revelations, we suggest that freezing-enhanced aminoglycoside uptake represents a direct or indirect effect of freezing injury on the cell membrane of bacteria.

**FIG 10:**
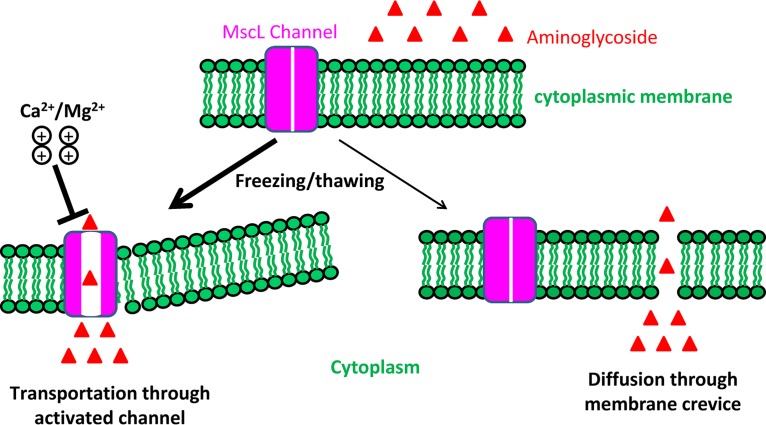
Freezing-induced membrane destabilization can activate the MscL channel, which in turn enhances aminoglycoside uptake. The key to the potentiation of aminoglycosides by freezing is to facilitate traversal of the cytoplasmic membrane of bacterial cells by positively charged aminoglycosides. Two conceivable mechanisms are diagrammed here. The first mechanism is transportation of aminoglycosides via ion channels (e.g., MscL), which could be caused by membrane destabilization as induced by the freezing/thawing treatments, and divalent cations such as Ca^2+^/Mg^2+^ can also be transported by the activated MscL and thus competitively inhibit aminoglycoside transportation. The second mechanism is diffusion of aminoglycosides through the crevices that are transiently formed in the destabilized cytoplasmic membrane. In light of our experimental data and observations from the literature, the MscL channel activation-based mechanism is the more likely of the two.

Furthermore, we propose that the freezing-enhanced aminoglycoside uptake is most likely accomplished by cytoplasmic membrane-localized ion channels such as MscL (as diagrammed in the left part of Fig. 10), based on several independent observations. First, E. coli *ΔmscL* cells exhibited a marginal increase in their resistance to the combined treatment (Fig. S8E). Second, only the complementary expression of the MscL channel, but not that of MscS, MscK, and YbdG channels, dramatically increased the sensitivity of MJF612 cells to the combined treatment (Fig. 8A). Third, the complementary expression of MscL, but not MscS, enabled MJF612 cells to take up much more streptomycin (Fig. 8B). Fourth, we found five physiological conditions under which the endogenously expressed MscL in E. coli cells was upregulated (Fig. 8C) and the sensitivity of the cells to the combined treatment of tobramycin and freezing was increased concomitantly (Fig. 8D; see also Fig. S9A to C). Last but not least, we observed a strong association between the sensitivity of the MJF612 cells to the combined treatment and the protein levels of the complementarily expressed MscL (Fig. 8E; see also Fig. S9D). Additional evidence supporting that interpretation of this MscL activation-based mechanism includes the following. First, the pore size of MscL is around 30 Å (72), which is large enough to allow aminoglycosides to flow in. Second, MscL activation does not require PMF and ATP, consistent with our observations. Collectively, our observations, in conjunction with studies from Blount and coworkers on the MscL-mediated uptake of dihydrostreptomycin under conventional treatment conditions (62, 63), strongly suggest that MscL directly mediates aminoglycoside uptake under freezing conditions.

MscL, a mechanosensitive ion channel, is well known to be activated in response to changes in membrane tension as elicited by osmotic shock, cold/heat shock stresses, and/or other types of mechanical forces (73–75). Conceivably, freezing leads to membrane damage and destabilization (Fig. 7) (48, 50, 53, 54), which, in turn, changes the membrane tension and thus activates MscL. Alternatively, freezing can directly result in osmotic shock (48, 52, 71), which, in turn, could also activate MscL (73, 74). It should be pointed out that besides MscL, other unidentified proteins, likely homologs of MscL, appear to also mediate the freezing-induced aminoglycoside potentiation in E. coli, given that the E. coli *ΔmscL* stationary-phase cells exhibited sensitivity comparable with that of the wild-type cells (Fig. S8D) and that the exponential-phase *ΔmscL* cells were still sensitive to the combined treatment (Fig. S8E).

Notably, the presence of Ca^2+^/Mg^2+^ significantly suppressed the MscL-enhanced aminoglycoside uptake (Fig. 8B) and potentiation (Fig. 9A) upon freezing. One may suspect that such suppression results from the effects of Ca^2+^ or Mg^2+^ on E. coli cells for protection from freezing-induced injury. Our cell survival assay revealed that neither Ca^2+^ nor Mg^2+^ protected E. coli cells from being killed by three cycles of freezing treatments (Fig. S9F). In addition, our SDS-PAGE analysis results show that neither Ca^2+^ nor Mg^2+^ suppressed the freezing-induced leakage of cellular proteins (Fig. S9G). Therefore, the suppressive effects of Ca^2+^ and Mg^2+^ more likely result from their direct actions on MscL. Given that Ca^2+^ can be transported from bacterial cells by the MscL channel (64, 76) and that MscL is able to nonspecifically transport ions to a certain degree (77), it is conceivable that Ca^2+^ and Mg^2+^ can also be transported into cells by freezing-activated MscL. As such, high (up to 10 mM) concentrations of Ca^2+^ and Mg^2+^ in cell suspensions would be able to competitively inhibit MscL-mediated aminoglycoside uptake (as diagrammed in Fig. 10) at a concentration of aminoglycoside of around 100 μM.

Another possible explanation for the freezing-induced aminoglycoside potentiation is that crevices or pores are formed in the cytoplasmic membrane of bacterial cells during the freezing/thawing treatment, such that aminoglycosides are able to freely diffuse through these crevices (as illustrated in the bottom right part of Fig. 10). This hypothesis is supported by the observations that freezing leads to (i) leakage of a large amount of cellular proteins (Fig. 7B) and other intracellular content (56–59) from the cells, (ii) increased permeability of the cell membrane (Fig. 7C; see also Fig. S8A), and (iii) cell membrane bending and wrinkles (Fig. 7D). Accordingly, this mechanism is PMF and ATP independent. Nevertheless, this mechanism cannot well explain why different types of aminoglycoside antibiotics with similar chemical structures and molecular weights differ in their bactericidal effects against one bacterium upon freezing (Table 1), assuming that their free diffusion rates through the membrane crevice would be basically equal. It also cannot explain why the presence of divalent cations (e.g., Ca^2+^ and Mg^2+^) can suppress the freezing-induced aminoglycoside uptake and potentiation. These contradictions, however, can be well explained by the MscL channel activation-based mechanism, which thus represents a more favorable hypothesis.

### Potential applications for freezing-induced aminoglycoside potentiation in eradicating multidrug-tolerant persisters.

Biological effects and the underlying mechanisms of freezing have long been investigated as an important aspect of cryobiology, which is a multidisciplinary science for the study of the physical and biological behaviors of cells and tissues at low temperatures (47, 48, 50, 51, 53). Advances in this field have facilitated the development of procedures for the preservation of cells of biological, medical, and agricultural significance (51) and for treating a number of diseases (e.g., skin conditions such as warts, moles, skin tags, and solar keratoses) (43, 44). Importantly, freezing has been utilized to treat skin, prostate, lung, and other types of cancers (43–46) (termed cryotherapy or cryosurgery). To the best of our knowledge, our study is the first report of its application in treating bacterial infections, possibly because the bactericidal effects of freezing alone on bacterial pathogens are weak (Fig. 1 and 4; see also Fig. S5) (71). A direct application of our combined treatment protocol to the eradication of persister bacterial cells would obviously be limited due to the inevitable freezing injury to animal cells and tissues. Nonetheless, our work is still of interest for developing promising antipersister strategies, based on the following considerations.

First, optimization of the cooling rate and the warming rate and the addition of appropriate cryoprotectants may minimize the freezing injury on the infected site of the animals or humans (48, 53) but meanwhile maintain the efficiency of the combined treatment in killing pathogen persisters, as implied by the results presented in Fig. 6. In particular, those cryoprotectants that can specifically protect animal cells from freezing injury but have no beneficial effect on pathogens would be ideal for this purpose. Second, if the precise biochemical mechanisms underlying the freezing-induced activation of MscL are unraveled, new strategies, other than freezing, for example, MscL activation with small-molecule agonists, might be developed for killing bacterial persisters. Notably, Blount and coworkers recently reported that MscL agonists are able to potentiate antibiotics against pathogens under conventional conditions (78), although their potentiation effects are weaker than those of freezing as reported here.

## MATERIALS AND METHODS

### Strains, culture conditions, and reagents.

Various Gram-negative (E. coli, P. aeruginosa, A. baumannii, K. pneumoniae, S. flexneri, and S. enterica serovar Typhimurium) and Gram-positive (S. aureus, B. subtilis, M. luteus, L. lactis, S. epidermidis, and E. faecalis) bacterial strains, E. coli strain MJF612 (Frag1 *ΔmscL*::cm, *ΔmscS*, *ΔmscK*::kan, and *ΔybdG*::*aprD*) and mechanosensitive ion channel mutants of E. coli (i.e., *ΔmscL*, *ΔmscS*, *ΔmscK*, *ΔmscM*, *ΔynaI*, *ΔybdG*, and *ΔybiO* mutants) were used in this study, and their characteristics are described in Table S1A in the supplemental material. Briefly, an overnight culture of each strain was diluted at 1:500 in Luria-Bertani (LB) medium (note that the MRS medium was used for L. lactis and E. faecalis) and agitated in a shaker (37°C, 220 rpm) for 3 to 6 h and 20 to 24 h to prepare exponential-phase and stationary-phase cells, respectively. The antibiotics used in our study included tobramycin, streptomycin, gentamicin, kanamycin, ampicillin, carbenicillin, ofloxacin, ciprofloxacin, chloramphenicol, rifampin, and erythromycin, and their manufacturers and final concentrations for different treatments are described in Table S1B. Carbonyl cyanide m-chlorophenyl hydrazone (CCCP) was purchased from Sigma-Aldrich and ^3^H-labeled tobramycin from American Radiolabeled Chemicals, Inc. (catalog no. ART1927, lot no. 160429). All other chemical reagents were of analytical purity.

### Treatment of bacteria with aminoglycosides and rapid freezing.

Exponential-phase (optical density at 600 nm [OD_600_] = 0.8) or stationary-phase cultures of E. coli, P. aeruginosa, and S. aureus cells were transferred to Eppendorf tubes (100 μl) and mixed thoroughly with each antibiotic at the final concentrations described in Table S1B and then immediately immersed in liquid nitrogen (–196°C) for 10 s or in prechilled ethanol (–80°C) for 20 s and subsequently thawed in an ice-water bath (with the procedure usually being completed within 10 min). For stationary-phase cells, one, two, or three rounds of freezing and thawing treatments were performed. Treated cells were washed twice using phosphate-buffered saline (PBS; 0.27 g/liter KH_2_PO_4_, 1.42 g/liter Na_2_HPO_4_, 8 g/liter NaCl, 0.2 g/liter KCl, pH 7.4) by centrifugation (13,000 × *g*, 30 s), and then 5 μl of 10-fold serially diluted cell suspension was spot plated onto LB agar dishes for survival assay. Other types of bacterial strains were treated similarly.

### Effects of freezing time, cooling rate, warming rate, and cryoprotectants on aminoglycoside potentiation.

To prolong the duration of the frozen state, the bacterium-containing Eppendorf tubes were immersed in liquid nitrogen or prechilled ethanol for various lengths of time (10 and 20 s and 1 and 3 min). Slow cooling was achieved by packaging the bacterium-containing PCR tube with an Eppendorf tube, which was additionally packed with a 15-ml plastic tube and then placed in a –80°C refrigerator for 20 min. Slow warming and rapid warming of the rapidly frozen cells at –196°C were achieved by thawing them in a 4°C refrigerator and in a 30°C water bath, respectively, and the bacterial cells were then immediately washed after full thawing that was usually completed within 15 and 1.5 min, respectively. The effects of the cryoprotectants were examined by mixing the cell cultures with glycerol and DMSO at final concentrations of 20% and 10%, respectively, before freezing.

### Preparation and eradication of persister and persister-like cells.

Persister cells were prepared by adding ampicillin (100 μg/ml) or ofloxacin (5 μg/ml) into the exponential-phase E. coli cells (OD_600_ of approximately 0.8) followed by continuous agitation for another 3 h according to a method described in earlier reports (32–34). Ofloxacin-tolerant P. aeruginosa persisters were prepared similarly by treating cells with ofloxacin (2.5 μg/ml). The cells were then concentrated 10-fold or 100-fold via centrifugation and resuspension, and they were subsequently thoroughly mixed with each type of aminoglycoside antibiotic at the concentrations indicated in Table S1B before being subjected to the rapid freezing treatment and survival assay as described above. For preparing persister-like cells, exponential-phase cultures of E. coli or P. aeruginosa cells were agitated with CCCP (20 μM), chloramphenicol (35 μg/ml), erythromycin (20 μg/ml), rifampin (100 μg/ml), or sodium azide (6 mM) at 37°C for 1 h and then mixed thoroughly with tobramycin (25 μg/ml). These cells were subjected to the rapid freezing treatment and survival assay with or without having been subjected to further agitation at 37°C for 1 h.

### Aminoglycoside pretreatment followed by rapid freezing.

Each type of aminoglycoside antibiotic was added to stationary-phase E. coli cell cultures at the concentrations indicated in Table S1B, and the cultures were continuously agitated at 37°C for 3 additional hours. The cell cultures were then transferred into Eppendorf tubes, and 1, 2, or 3 cycles of rapid freezing (in liquid nitrogen) and thawing (in ice water) were performed before bacterial survival assay as described above. Exponential-phase P. aeruginosa cell cultures were supplemented with tobramycin (12.5 μg/ml), continuously agitated at 37°C for 1, 2, or 3 h, transferred into Eppendorf tubes, and subjected to the rapid freezing treatment and survival assay.

### Aminoglycoside uptake assay.

The bacterial uptake of tobramycin was measured by two independent methods. In the radioactivity assay, a stock solution of 15 μCi ^3^H-labeled tobramycin/ml was prepared in pure water and stored at –20°C. Referring to the method described in our earlier report for assaying ^3^H-labeled estrogen uptake in E. coli cells (79), exponential-phase E. coli cell cultures were concentrated 4-fold by centrifugation and resuspension, thoroughly mixed with ^3^H-labeled tobramycin at a final concentration of 0.015 μCi per 100 μl, and then subjected to the rapid freezing treatment. After thawing in an ice-water bath, the cells were centrifuged (16,000 rpm, 15 s), washed once with the PBS buffer, resuspended in a lysis buffer (0.2 M NaOH, 1% SDS), and incubated at 90°C for 30 min. The cell lysates were then cooled, centrifuged quickly (800 rpm, 10 s), and mixed with a 5-volume scintillation cocktail before subjected to radioactivity measurement on a PerkinElmer Tri-Carb3110TR liquid scintillation analyzer. The nonradioactivity assay was performed as recently described by us (41), such that tobramycin as taken up by E. coli cells was extracted by cell lysis and then dropped on LB agar dishes to inhibit bacterial cell growth.

### Membrane integrity analyses.

**(i) NPN staining.** The integrity of the outer membrane of E. coli cells after the freezing treatment was analyzed as we previously reported (80) by recording the fluorescence intensity of NPN (1-N-phenylnaphthylamine), which increases upon interacting with the hydrophobic tail of membrane phospholipids (55). Briefly, exponential-phase E. coli cells were resuspended in 5 mM HEPES buffer (pH 7.2), with or without the addition of 25 μg/ml tobramycin, and then subjected to freezing in liquid nitrogen and thawing in an ice-water bath before being mixed with acetone-dissolved NPN (to reach a final concentration of 10 μM). The fluorescence intensity was recorded with a Hitachi F-4600 fluorescence spectrophotometer at the excitation and emission wavelengths of 350 and 420 nm, respectively.

**(ii) Protein leakage.** The integrity of the cell membrane after the freezing treatment was evaluated by examining the cellular protein leakage into the medium. For this, stationary-phase E. coli cells were resuspended in the PBS buffer, mixed with tobramycin (100 μg/ml), and then subjected to the freezing treatment in liquid nitrogen and thawing in an ice-water bath three times before centrifugation at 10,000 × *g* for 2 min. Proteins that leaked into the surrounding medium were concentrated by precipitation with 10% trichloroacetic acid, washed with acetone, and then analyzed using 10% SDS-PAGE (visualized by Coomassie blue staining).

**(iii) Propidium iodide (PI) staining.** Exponential-phase E. coli cells (100 μl) were frozen in the absence or presence of 25 μg/ml tobramycin and then washed and incubated at room temperature in PBS–propidium iodide (PI) (50 μg/ml) for 10 min. Samples were analyzed on a BD Accuri C6 flow cytometer with the following settings: detector, FL2; flow rate, 14 μl/min; core size, 10 μm. Meanwhile, the treated cells were plotted on agarose after PI staining. Images were recorded at 30°C with an N-SIM imaging system (Nikon) equipped with a 100×/1.49-numerical-aperture (NA) oil-immersion objective (Nikon) and laser beams (561 nm).

**(iv) Electron microscopy analysis.** Exponential-phase E. coli cells (100 μl) were frozen in the absence or presence of 25 μg/ml tobramycin, treated with sucrose (with a final concentration of 20%) for 1 min, fixed with glutaraldehyde and formaldehyde (at final concentrations of 0.1% and 2%, respectively) for 1 h, and subjected to postfixation and dehydration. Cells were subjected to ultrathin sectioning (using a PowerTome-XL ultramicrotome) and negatively stained with 2% uranyl acetate before being imaged on a JEM-1400 transmission electron microscope operated at 80 kV at a magnification of 3,000 or 25,000.

### Assay of intracellular ATP levels in E. coli exponential-phase cells.

A luciferase-based kit (catalog no. S0026B; Beyotime Biotechnology, Shanghai, China) was used to measure ATP levels according to the manufacturer’s instructions. Briefly, E. coli exponential-phase cells, with or without pretreatment of 20 μM CCCP or 6 mM NaN_3_ at 37°C for half an hour, were lysed using the lysis buffer and then centrifuged (12,000 × *g*, 4°C, 5 min). The supernatant was quickly mixed with the working solution at equal volumes and then transferred into a 96-well plate before light recording on a FLUOstar Omega microplate reader was performed using the Luminometer method. Cells treated with rapid freezing for 10 s with liquid nitrogen were also analyzed.

### Proton motive force assay.

A flow cytometry-based assay was applied to measure the proton motive force by using the fluorescence probe 3,3′-diethyloxacarbocyanine iodide [DiOC2(3)] (purchased from MaoKang Biotechnology, Inc., Shanghai, China) according to the manufacturer’s instructions. Briefly, E. coli exponential-phase cells, with or without CCCP pretreatment for 1 h, were diluted into PBS to a cell density of 10^6^ cells/ml and incubated with DiOC2(3) (at a final concentration of 30 μM) at room temperature for 15 min. Cells were subjected to flow cytometric analysis on a FACSymphony A5 system (BD Biosciences) with excitation at 488 nm and emission in both the red (630-nm) channel and green (515-nm) channel. Cells treated by rapid freezing for 10 s with liquid nitrogen were also analyzed.

### Animal experiments.

Eight-week-old ICR male mice (around 28 g in body weight) were purchased from the Animal Center of Fujian Medical University and maintained in the Animal Center of Fujian Normal University. Mice were housed for 1 or 2 days and then randomly divided into four groups for surgery experiments (group A, room temperature without tobramycin; group B, room temperature with tobramycin; group C, freezing without tobramycin; group D, freezing with tobramycin; *n* = 3). Briefly, after anesthetization by intraperitoneal injection of 4% chloral hydrate, the tails of the mice were sterilized using 70% ethanol, and 30-μl stationary-phase cultures of P. aeruginosa cells premixed with 100 μg/ml tobramycin were seeded on the tails. After full absorption and drying, the mice tails were wrapped with Parafilm and then immersed in liquid nitrogen for 2 min. The mice were sacrificed half an hour later, and the tails were cut and homogenized in 2 ml PBS buffer, with the lysates being spot plated on LB agar dishes for bacterial survival assay.

The muscle of the sacrificed mice from group A was cut and placed in Eppendorf tubes for conducting the *in situ* freezing studies. Briefly, 5 μl tobramycin-containing stationary-phase cultures of the P. aeruginosa cells were spot plated on these tissues and then subjected to freezing treatment in liquid nitrogen for 1 min. After addition of 2 ml PBS buffer, the mixtures were shaken for 5 min and the remaining supernatant was spot plated on LB agar dishes for bacterial survival assay.

In addition, we applied a skin acute wound model to evaluate the efficacy of the combined treatment. Briefly, mice were anesthetized, barbered on the right back, and sterilized, and then a 1-cm-by-1-cm whole-skin section was removed to make an acute skin wound according to a method described in an earlier report (49). Then, 5 μl of 10-fold-concentrated exponential-phase P. aeruginosa cells (with or without CCCP or chloramphenicol pretreatment) was seeded on the wound and allowed to fully absorb before addition of 40 μl cell-free culture medium (i.e., cells were removed by centrifugation) containing 50 or 100 μg/ml tobramycin. After full drying, mice were locally frozen at the wound site by using liquid nitrogen-absorbed medical cotton and then the wound site was bandaged firmly with medical gauze. After mice were housed overnight, the whole scab on the wound site was removed and homogenized, with the lysates being spot plated on LB agar dishes for bacterial survival assay. For quantification, each sample was spot plated in triplicate.

### Ethics statement.

The animal use protocol was approved by the Animal Ethical and Welfare Committee of Fujian Normal University (approval no. IACUC 20190006), and the animal experiments were performed in accordance with the National Standards of the People’s Republic of China (GB/T 35892-2018, laboratory animals—guideline for ethical review of animal welfare; GB/T 35823-2018, laboratory animals—general requirements for animal experiment).

### Complementary expression of mechanosensitive ion channels.

pCA24N plasmids for use in expressing the MscL, MscS, MscK, and YbdG channels were picked from the ASKA library (81), and the chloramphenicol-resistant gene in pCA24N was replaced by the ampicillin-resistant gene (as cloned from the pBAD plasmid), with the new plasmid being designated pCA24NA. E. coli strain MJF612 was transformed with these plasmids, cultured in LB medium to an OD_600_ of 0.6, and induced to express ion channels by incubation with 0.5 mM isopropyl β-d-1-thiogalactopyranoside (IPTG) for 1 h before being subjected to the combined treatment of streptomycin plus freezing as described above. In particular, the complementary expression of MscL was induced with elevated concentrations of IPTG (1 μM, 5 μM, 10 μM, and 1 mM).

### Detection of endogenously expressed MscL in E. coli cells.

To detect the endogenously expressed MscL protein in E. coli BW25113 cells, the *mscL* gene in the genome was modified by the use of a CRISPR-Cas system according to a method described in an earlier report (82) using GAACGTGGTGGATTTGGCGG as the N20 primer. At the end, a segment of DNA sequence (GGCAGCGGCCTGAACGATATTTTTGAAGCGCAGAAAATTGAATGGCATGAA) was inserted into the *mscL* gene before the TAA stop codon such that an Avi tag was added to the C-terminal end of the MscL protein, and the resulting strain was named BW25113-MscL_avi_. This strain was cultured in LB medium, NaCl-free LB medium, M9 medium plus 5 g/liter glucose medium or MHB medium. In addition, nutrient shift-induced and starvation-induced persisters were prepared as we previously described (41) and then treated with osmotic shock for 10 min using 1.25 M sucrose. All these cells were adjusted to an OD_600_ of 0.6 and then subjected to the combined treatment with 25 μg/ml tobramycin plus freezing in –80°C ethanol twice before cell survival assay. Meanwhile, cell lysates were separated with SDS gel and the Avi-tagged MscL protein was probed by the use of alkaline phosphatase-conjugated streptavidin (which binds to the biotin attached to the Avi tag).

### Statistics.

CFU from LB agar dishes were counted during cell survival assays, and cell density was calculated according to the dilution fold and the volume of the cell droplet. Error bars, as calculated by the use of Microsoft Excel, represent means ± standard deviations (SD) of results from triplicate samples. At least two independent experiments were performed for all experiments. A statistical analysis was performed using MicroOrigin software with the analysis of variance (ANOVA) algorithm at a significance level of 0.05.
