# Supplementary material for: Rapid Freezing Enables Aminoglycosides To Eradicate Bacterial Persisters via Enhancing Mechanosensitive Channel MscL-Mediated Antibiotic Uptake
Source: mBio. 2020 Feb 11;11(1):e03239-19. doi: 10.1128/mBio.03239-19 (PMC7018644; doi:10.1128/mBio.03239-19)
Supplement: FIG S1 [file mBio.03239-19-sf001.pdf]

Figure S1

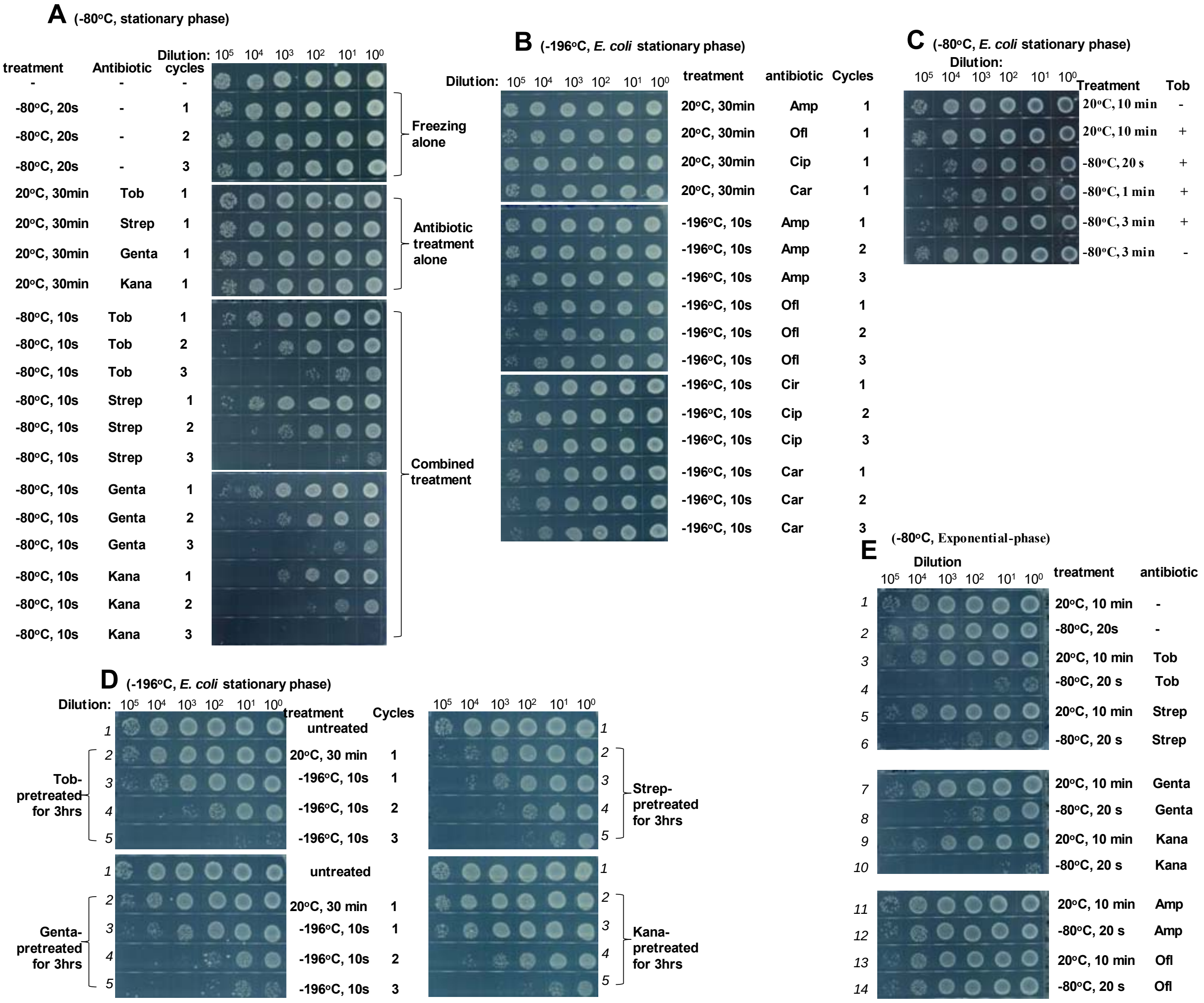

Fig. S1 Aminoglycoside potentiation by freezing against *E. coli* cells.

(**A, B**) Survival of stationary-phase *E. coli* cells on LB agar dishes after the cells were mixed with the indicated antibiotics and subjected to cycled freezing in ethanol pre-chilled at -80ºC for 20 sec (Panel A) or in liquid nitrogen for 10 sec (Panel B) and thawing in ice-water. (**C**) Survival of stationary-phase *E. coli* cells on LB agar dishes after the cells were frozen in ethanol pre-chilled at -80ºC for 20 sec, 1 min or 3 min in the presence of 100 µg/mL tobramycin. (**D**) Survival of stationary-phase *E. coli* cells on LB agar dishes after the cells were agitated at 37ºC for 3 hours in presence of the indicated antibiotics and then subjected to cycled freezing in liquid nitrogen for 10 sec and thawing in ice-water. Tob: tobramycin; Strep: streptomycin; Genta: gentamicin; Kana: kanamycin; Amp: ampicillin; Car: Carbenicillin; Ofl: ofloxacin. Cip: Ciprofloxacin. Concentrations of antibiotics for treatment are described in **Table S1B**. (**E**) Survival of exponential-phase *E. coli* cells (OD_{600}≈0.8) on LB agar dishes after the cells were mixed with the indicated antibiotics and then subjected to freezing in ethanol pre-chilled at -80ºC for 20 sec and thawing in ice-water bath. Tob: tobramycin; Strep: streptomycin; Genta: gentamicin; Kana: kanamycin; Amp: ampicillin; Ofl: ofloxacin. Concentrations of antibiotics for treatment are described in **Table S1B**.
