# Supplementary material for: Rapid Freezing Enables Aminoglycosides To Eradicate Bacterial Persisters via Enhancing Mechanosensitive Channel MscL-Mediated Antibiotic Uptake
Source: mBio. 2020 Feb 11;11(1):e03239-19. doi: 10.1128/mBio.03239-19 (PMC7018644; doi:10.1128/mBio.03239-19)
Supplement: TABLE S1 [file mBio.03239-19-st001.docx]

**Table S1A Bacterial strains used in this study**

| **Bacterial strains** | **Origins** | **Characteristics** |
| --- | --- | --- |
| *Escherichia coli* BW25113 | Purchased from the Nara Institute of Science and Technology (Ikoma, Nara, Japan) | G^-^, the parent strain (wild type) for Keio collection |
| *E. coli* MJF612 strain | A gift from Prof. Paul Blount at University of Texas Southwestern Medical Center | MJF612 (Frag1 *ΔmscL*::cm, *ΔmscS*, *ΔmscK*::kan, *ΔybdG*:: aprD) |
| *Pseudomonas aeruginosa* PAO1 | A gift from Dr. Zhexian Tian at Peking University | G^-^, kanamycin- and ampicillin-resistant |
| *Acinetobacter baumanii* Ab6 | A gift from Prof. Xuanxian Peng at Sun Yat-Sen University | G^-^, multi-drug resistant |
| *Klebsiella pneumoniae* KP-D367 | The same as above | G^-^, multi-drug resistant |
| *Salmonella typhimurium* SL1344 | A gift from Prof. Xiaoyun Liu at Peking University | G^-^, Streptomycin-resistant |
| \| *Shigella flexneri* 24T7T \| \| --- \| | The same as above | G^-^, Streptomycin-resistant |
| *Staphylococcus aureus ATCC25923* | A gift from Prof. Luhua Lai at Peking University | G^+^ |
| [*Lactococcus lactis*](http://www.bnbio.com/p_5/p_195305.html) *NZ9000* | A gift from Dr. Qingeng Huang at Fujian Normal University | G^+^, multi-drug resistant |
| *Enterococcus faecalis ATCC29212* | Purchased from Hangzhou Binhe Microorganism Reagent Co, Ltd. | G^+^, multi-drug resistant |
| *Bacillus subtilis* | A gift from Prof. Baoyu Tian at Fujian Normal University | G^+^ |
| *Staphylococcus epidermidis CMCC26069* | Purchased from Hangzhou Binhe Microorganism Reagent Co, Ltd. | G^+^, Streptomycin-resistant |
| *Micrococcus luteus CMCC28001* | A gift from Dr. Qingeng Huang at Fujian Normal University | G^+^, tobramycin- and kanamycin-resistant |

**Table S1B Antibiotics and their used concentrations**

| Antibiotics | Suppliers | For exponential-phase cells (μg/mL) | For stationary-phase cells (μg/mL) |
| --- | --- | --- | --- |
| Ampicillin | Beijing Solarbio Science & Technology Co., Ltd. | 100 | 200 |
| Carbenicillin | Sangon Biotech (Shanghai) Co., Ltd. | 50 | 200 |
| Ofloxacin | Beijing Solarbio Science & Technology Co., Ltd. | 2.5 or 5 *^a^* | 20 |
| Ciprofloxacin | Sangon Biotech (Shanghai) Co., Ltd. | 5 | 20 |
| Tobramycin | Sangon Biotech (Shanghai) Co., Ltd. | 12.5, 25, 50 or 100 *^b^* | 100 |
| Kanamycin | Sangon Biotech (Shanghai) Co., Ltd. | 50 | 200 |
| Gentamicin | Sangon Biotech (Shanghai) Co., Ltd. | 25 | 100 |
| Streptomycin *^c^* | Beijing Solarbio Science & Technology Co., Ltd | 100 | 400 |
| Chloramphenicol | Sangon Biotech (Shanghai) Co., Ltd. | 35 | - |
| Erythromycin | Aladdin Industrial Corporation (Shanghai) | 20 | - |
| Rifampicin | Beijing Solarbio Science & Technology Co., Ltd | 100 | - |

*^a^* *P. aeruginosa* and *E. coli* persisters were prepared by treating the exponential-phase cells with ofloxacin at concentrations of 2.5 and 5 μg/mL, respectively.

*^b^* *E. coli* mutant strains were treated with tobramycin at a concentration of 12.5μg/mL (refer to **Figs. 7E, S8C**); Exponential-phase *P. aeruginosa* cells were treated with tobramycin at a concentration of 12.5 μg/mL (refer to **Fig. 4A**); For other bacterial strains, tobramycin at a concentration of 25 μg/mL was used; for animal experiments, tobramycin at concentrations of 50 or 100 μg/mL were used.

*^c^* The sensitivity of single channel-deletion mutants and *E. coli* MJF612 mutant cells complementarily expressing each channel was tested using streptomycin at varying concentrations (50, 100 and 200 μg/mL in **Figs. 8A** and **S8E**; 200, 400 and 1000 μg/mL in **Fig. S8D**).
