# Supplementary material for: Rapid Freezing Enables Aminoglycosides To Eradicate Bacterial Persisters via Enhancing Mechanosensitive Channel MscL-Mediated Antibiotic Uptake
Source: mBio. 2020 Feb 11;11(1):e03239-19. doi: 10.1128/mBio.03239-19 (PMC7018644; doi:10.1128/mBio.03239-19)
Supplement: FIG S2 [file mBio.03239-19-sf002.pdf]

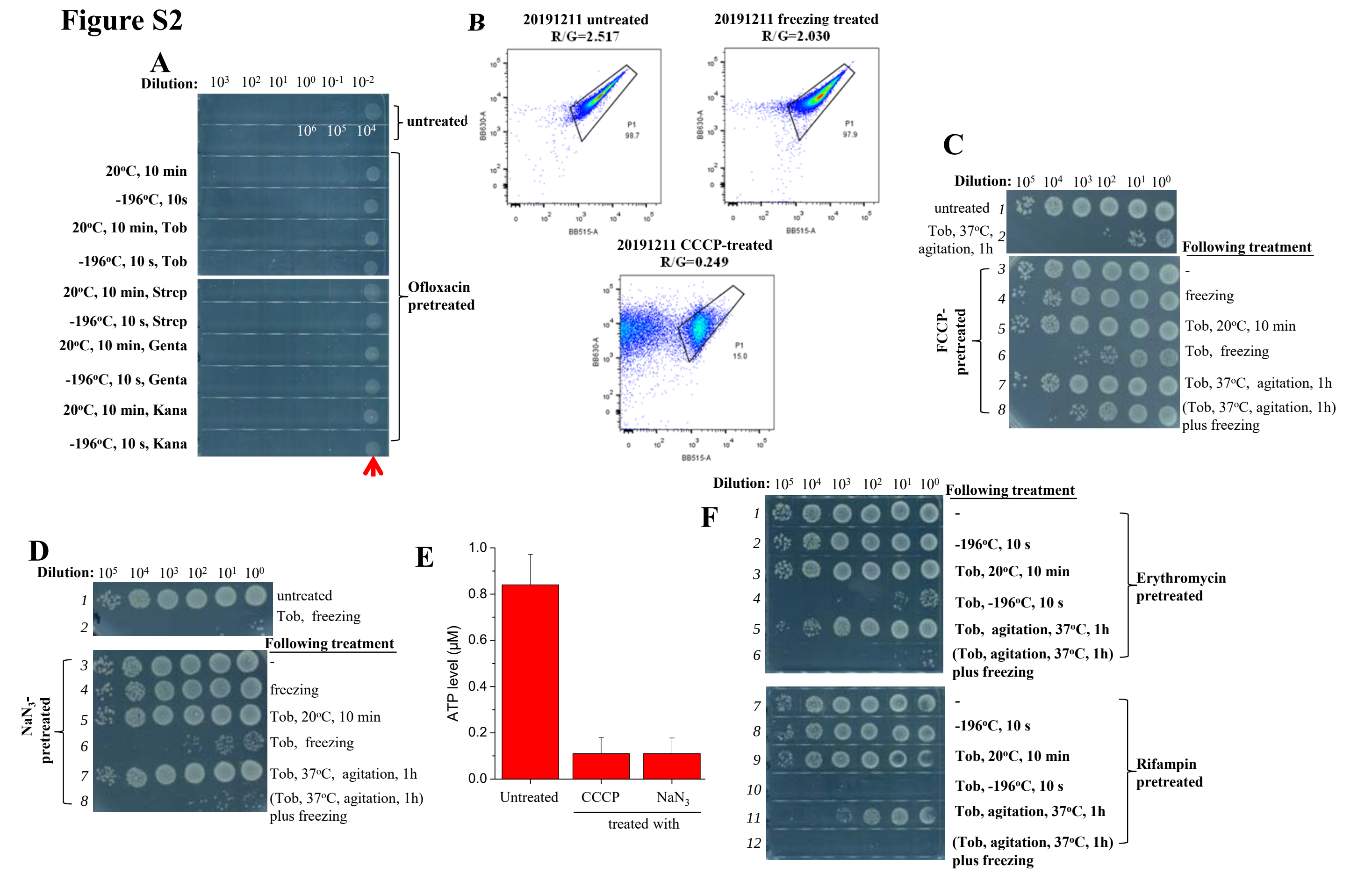

Fig. S2 Freezing facilitates aminoglycosides to eradicate antibiotic-tolerant *E. coli* persister-like cells independently of PMF

(**A**) Pre-growth state of ofloxacin-pretreated exponential-phase *E. coli* cells on LB agar dishes. Cells were pre-treated with 5 μg/mL ofloxacin at 37°C for three hours, concentrated at 100-fold (i.e., a dilution of 10^{-2}), mixed with indicated aminoglycoside and then subjected to freezing/thawing treatment before bacterial survival assay. The photo of the LB agar dish was taken before incubation for cell growth. Note: 5 μl of the 100-fold concentrated cells after being plated on the dish are visible due to the extremely high cell density (as indicated by the red arrow). The result for the survival of these cells is shown in **Fig. 2B**. (**B**) Results of a flow cytometric analysis on the PMF of *E. coli* exponential-phase (OD_{600}≈0.55) following freezing or CCCP pretreatment. Cells at a density of 10^6 cells/mL were incubated with the membrane potential fluorescence probe DiOC2(3) before analysis. The ratio of red fluorescence to green fluorescence is designated on the top of each graph. (**C, D, F**) Survival of *E. coli* persister-like cells on LB agar dishes. Exponential-phase *E. coli* cells were agitated with 20 μM FCCP (Pane C), 6 mM sodium azide (Panel D), or 20 μg/mL erythromycin or 100 μg/mL rifampicin at 37°C (Panel F) for one hour followed by the combined treatments with 25 μg/mL tobramycin and freezing. Pre-treated cells were also mixed with tobramycin and agitated at 37°C for one hour (line 7 in Panels C and D; lines 5 and 11 in Panel F) before freezing (line 8 in Panels C and D; lines 6 and 12 in Panel F). (**E**) ATP concentrations of cell lysates of *E. coli* exponential-phase cells, which were pretreated with 20 μM CCCP or 6 mM NaN_3 for half an hour before lysis and luciferase assay. Data represent mean ± SD from three replicates.
