# Supplementary material for: Rapid Freezing Enables Aminoglycosides To Eradicate Bacterial Persisters via Enhancing Mechanosensitive Channel MscL-Mediated Antibiotic Uptake
Source: mBio. 2020 Feb 11;11(1):e03239-19. doi: 10.1128/mBio.03239-19 (PMC7018644; doi:10.1128/mBio.03239-19)
Supplement: FIG S3 [file mBio.03239-19-sf003.pdf]

## Figure S3

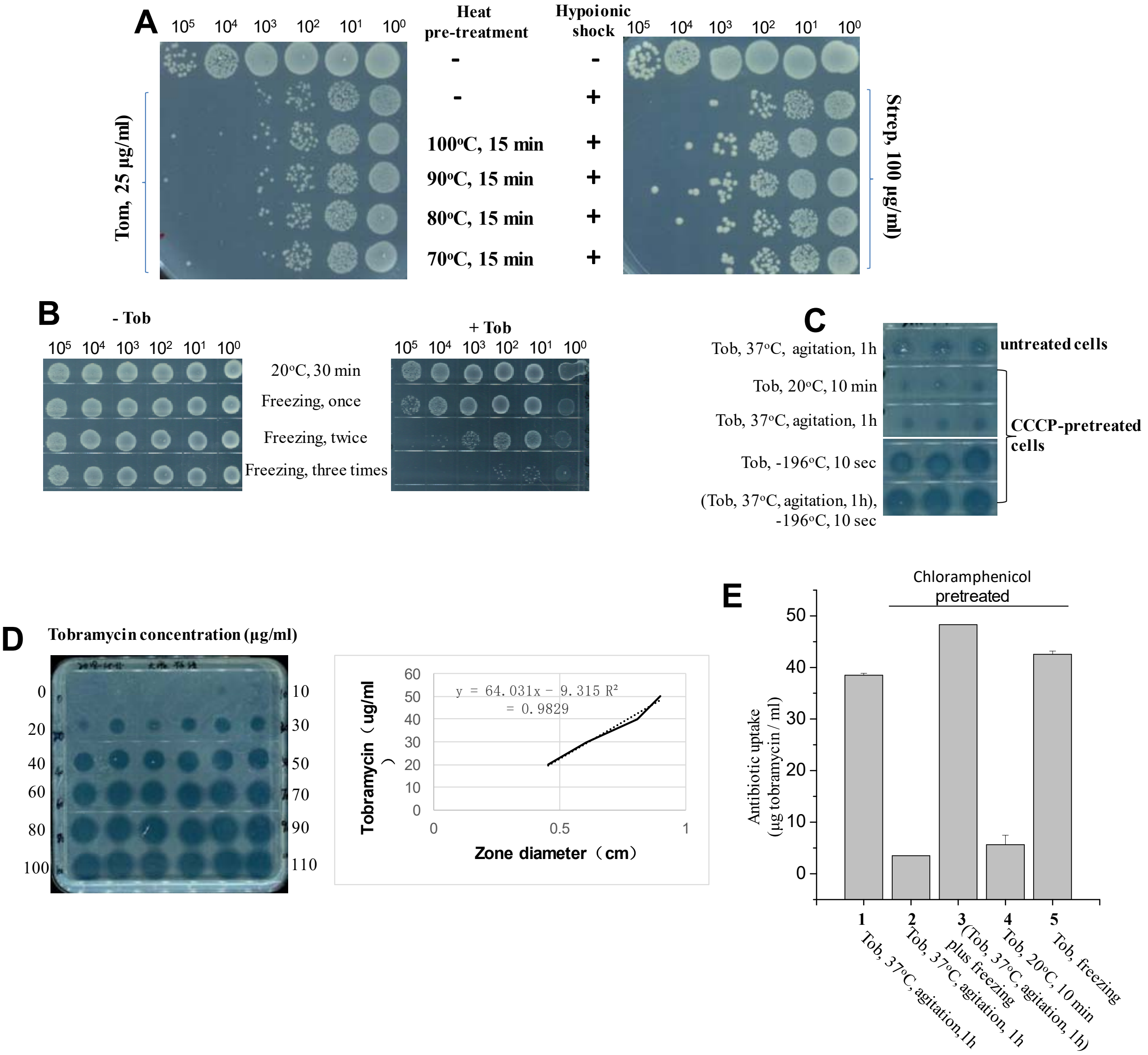


**Fig. S3 Freezing enhances the bacterial uptake of tobramycin.**

(**A**) Survival of stationary-phase *E. coli* cells on LB agar dishes after the cells were subjected to hypoionic shock in the presence of 25 μg/mL tobramycin (left) or 200 μg/mL streptomycin (right) for 2 min by referring to our earlier report [1], with the tobramycin- or streptomycin-containing water being pre-heated at the indicated temperatures. This result shows their high thermal stability and guides us to perform antibiotic extraction experiments by thermal denaturing the cell lysates at 90ºC for 15 min to remove cellular proteins but maintain the antibiotic activity. (**B**) Survival of stationary-phase *E. coli* cells on LB agar dishes after the cells were mixed with tobramycin and subjected to cycled freezing in liquid nitrogen for 10 sec and thawing in ice-water. Tobramycin accumulated in these cells was extracted and then subjected to cell growth inhibition assay, the results of which are shown in **Fig. 3B**. (**C**) Growth inhibition of *E. coli* cells on LB agar dishes by tobramycin extracted from CCCP-pretreated exponential-phase *E. coli* cells undergoing the indicated treatment. The diameter of each inhibition zone was measured and used for quantifying the level of tobramycin uptake. (**D**) Left part: growth inhibition of *E. coli* cells on LB agar dishes by tobramycin extracted from the mixture of exponential-phase *E. coli* cells and tobramycin, with the antibiotic being directly added into the lysozyme-containing cell wall-digestion buffer at the indicated concentrations. The diameter of each inhibition zone was measured and used for regression analysis to obtain the standard curve as presented in the right part. (**E**) Quantification of tobramycin accumulation in chloramphenicol-pretreated exponential-phase *E. coli* cells. Cells were treated as described in **Fig. 2C** and then subjected to tobramycin extraction. Data represent mean ± SD from three replicates.
