# Supplementary material for: Rapid Freezing Enables Aminoglycosides To Eradicate Bacterial Persisters via Enhancing Mechanosensitive Channel MscL-Mediated Antibiotic Uptake
Source: mBio. 2020 Feb 11;11(1):e03239-19. doi: 10.1128/mBio.03239-19 (PMC7018644; doi:10.1128/mBio.03239-19)
Supplement: FIG S4 [file mBio.03239-19-sf004.pdf]

Figure S4

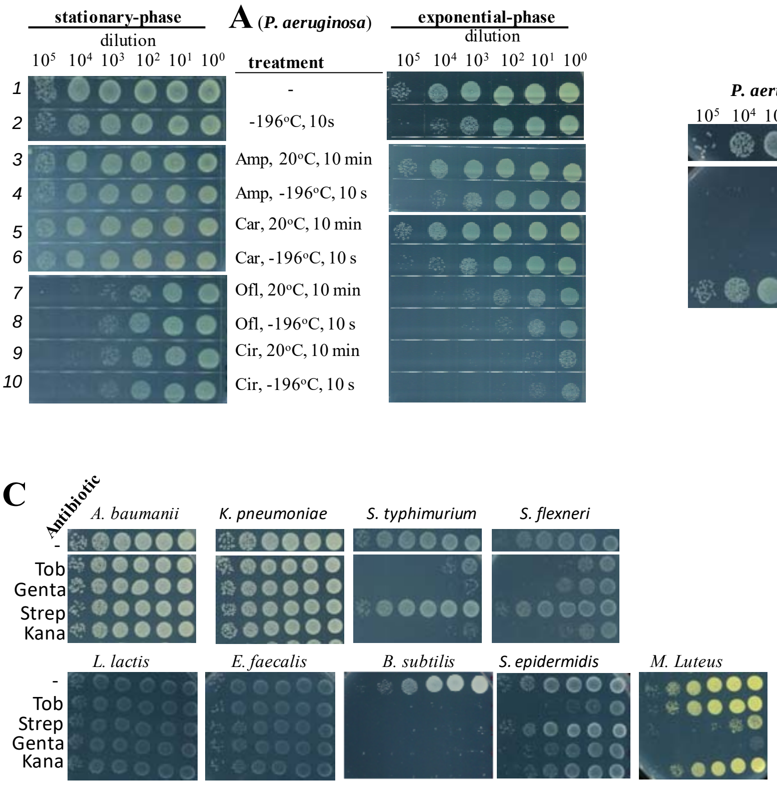

Fig. S4 Antibiotic sensitivity of various bacterial strains.

(**A**) Survival of stationary- and exponential-phase *Pseudomonas aeruginosa* cells on LB agar dishes after the cells were mixed with the indicated antibiotics and subjected to freezing in liquid nitrogen for 10 sec and thawing in ice-water. Amp: ampicillin; Car: Carbenicillin; Ofl: ofloxacin. Cip: Ciprofloxacin. Note: the decrease in the viability as observed for exponential-phase cells treated with ampicillin or carbenicillin plus freezing (lines 4 and 6 in the right part) should be considered as a result from freezing itself rather than a synergistic effect between freezing and aminoglycoside, given that the exponential-phase *P. aeruginosa* cells are moderately sensitive to freezing (line 2, right part of **Fig. 4A**). (**B**) Antibiotic sensitivity test of exponential-phase *Pseudomonas aeruginosa* and *Staphylococcus aureus* cells on LB agar dishes after the cells were agitated in presence of the indicated antibiotics at 37°C for two hours. The results show the high tolerance of *P. aeruginosa* cells to kanamycin and the relative high sensitivity of *S. aureus* cells to tobramycin. Tobramycin was thus chosen for the combined treatment experiment (refer to **Fig. 4B**). (**C**) Antibiotic sensitivity test of exponential-phase cells of the indicated Gram-negative (upper part) and Gram-positive (lower part) bacteria on LB as performed on LB agar dishes after the cells were mixed with the indicated antibiotics and agitated at 37°C for two hours. Tob: tobramycin; Strep: streptomycin; Genta: gentamicin; Kana: kanamycin. Concentrations of antibiotics for treatment are described in **Table S1B**.
