# Supplementary material for: Rapid Freezing Enables Aminoglycosides To Eradicate Bacterial Persisters via Enhancing Mechanosensitive Channel MscL-Mediated Antibiotic Uptake
Source: mBio. 2020 Feb 11;11(1):e03239-19. doi: 10.1128/mBio.03239-19 (PMC7018644; doi:10.1128/mBio.03239-19)
Supplement: FIG S5 [file mBio.03239-19-sf005.pdf]

Figure S5

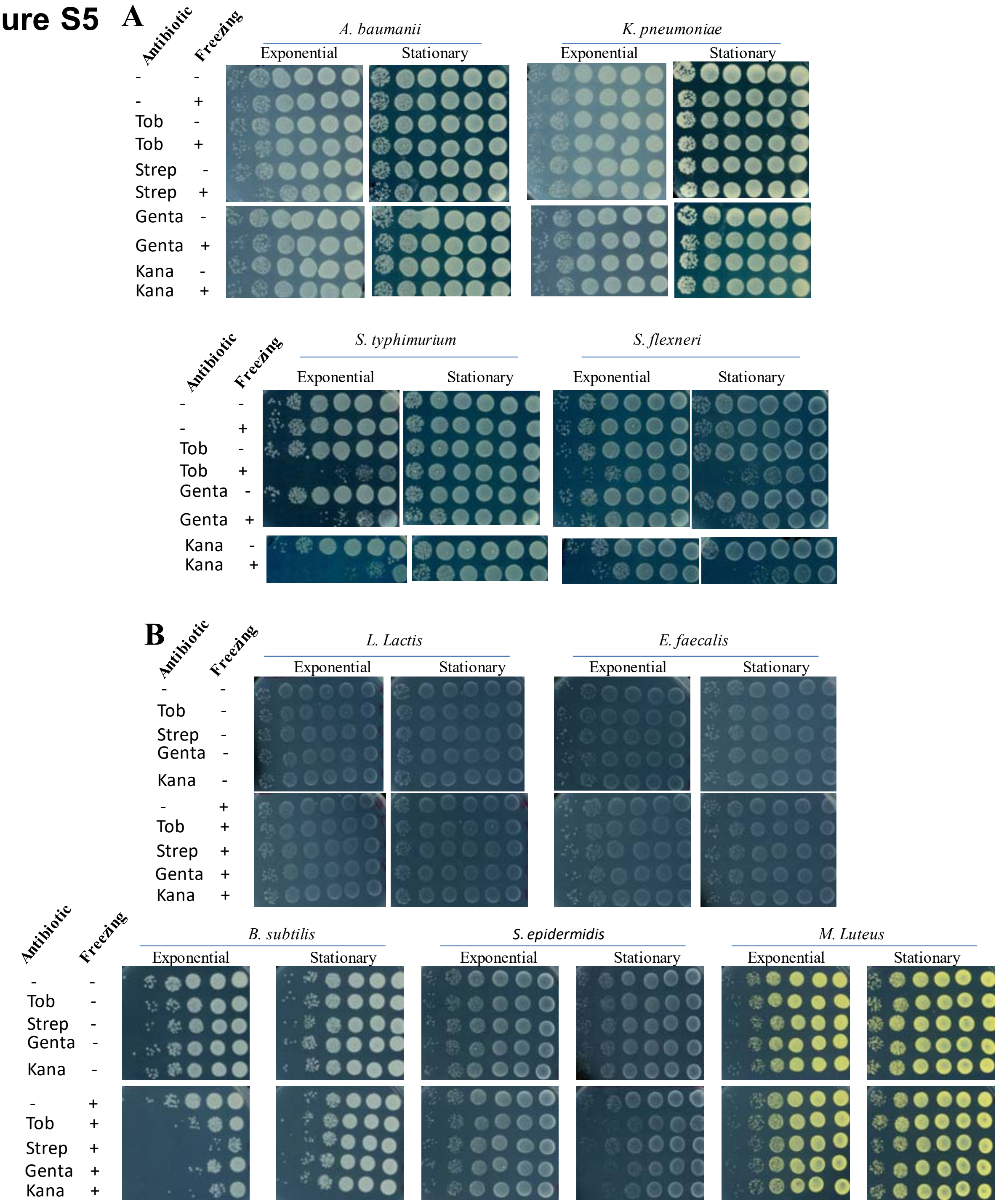

Fig. S5 Freezing potentiates aminoglycosides against exponential- and/or stationary-phase cells of several bacterial strains.

(**A, B**) Survival of stationary-phase and exponential-phase cells of the indicated Gram-negative (**A**) and Gram-positive (**B**) bacteria as performed on LB agar dishes after the cells were mixed with the indicated antibiotics and subjected to freezing in liquid nitrogen for 10 sec and thawing in ice-water. For stationary-phase cells, the freezing/thawing treatment was repeatedly performed for three times. For *S. typhimurium* and *S. flexneri*, streptomycin was not tested due to the resistance of these two bacteria.
