# Supplementary material for: Rapid Freezing Enables Aminoglycosides To Eradicate Bacterial Persisters via Enhancing Mechanosensitive Channel MscL-Mediated Antibiotic Uptake
Source: mBio. 2020 Feb 11;11(1):e03239-19. doi: 10.1128/mBio.03239-19 (PMC7018644; doi:10.1128/mBio.03239-19)
Supplement: FIG S6 [file mBio.03239-19-sf006.pdf]

Figure S6

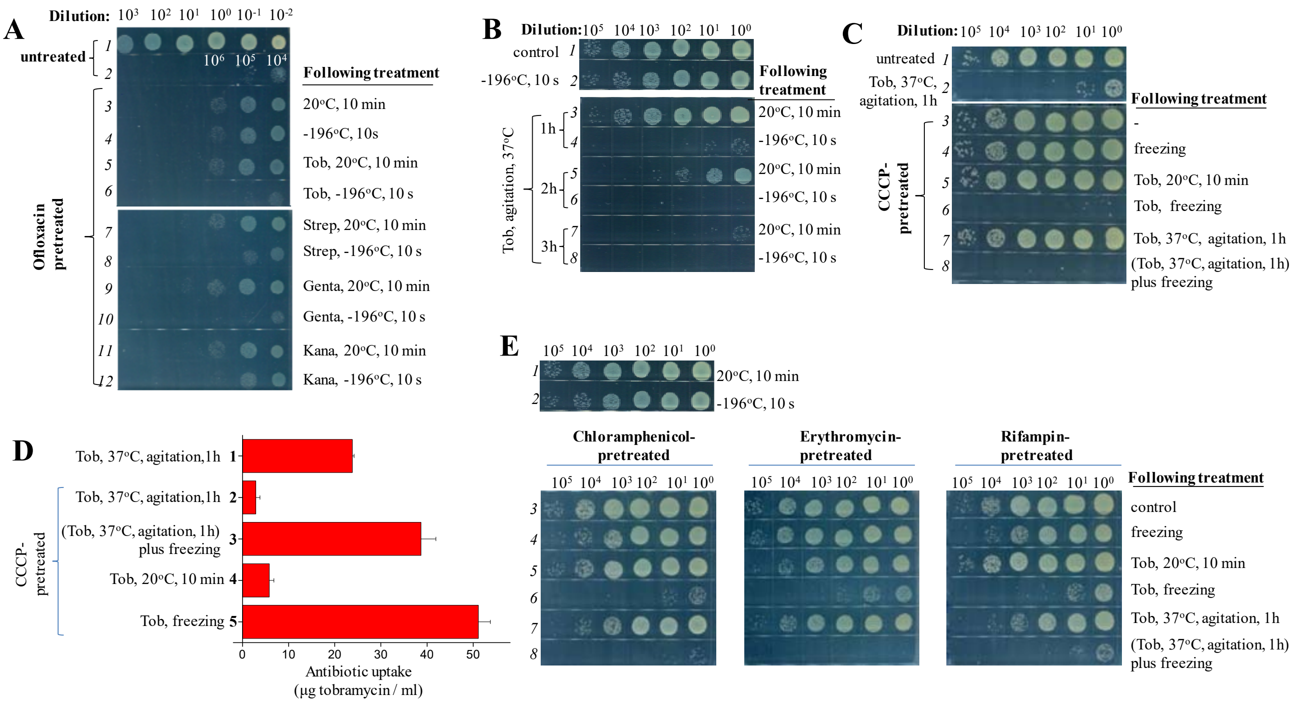

Fig. S6 Freezing facilitates aminoglycosides to kill *P. aeruginosa* persisters independently of PMF

(**A**) Survival of ofloxacin-tolerant *P. aeruginosa* persisters on LB agar dishes. Exponential-phase *P. aeruginosa* cells were pre-treated with 2.5 µg/mL ofloxacin at 37°C for three hours, 100-fold concentrated and subsequently subjected to the indicated combined treatment of aminoglycoside and freezing before survival assay. (**B**) Survival of tobramycin-tolerant *P. aeruginosa* persisters on LB agar dishes. Exponential-phase *P. aeruginosa* cells were pre-treated with 12.5 µg/mL tobramycin at 37°C for the indicated durations and then subjected to freezing before survival assay. (**C**) Survival of *P. aeruginosa* persister-like cells on LB agar dishes. Exponential-phase *P. aeruginosa* cells were pre-treated with 20 µM CCCP at 37°C for one hour followed by the combined treatment with 25 µg/mL tobramycin and freezing (line 6). Pre-treated cells were also mixed with tobramycin and agitated at 37°C for one hour (line 7) before freezing (line 8). (**D**) Quantification of tobramycin uptake in CCCP-pretreated exponential-phase *P. aeruginosa* cells. Cells were treated as described in Panel C, and tobramycin was extracted from the treated cells and subjected to the cell growth inhibition assay. Data represent mean ± SD from three replicates. (**E**) Survival of exponential-phase *P. aeruginosa* cells on LB agar dishes after the cells were pre-treated with 35 µg/mL chloramphenicol, 20 µg/mL erythromycin or 100 µg/mL rifampicin at 37°C for one hour followed by the combined treatment with 25 µg/mL tobramycin plus freezing (line 6). Pre-treated cells were also mixed with tobramycin and agitated at 37°C for one hour (line 7) before freezing (line 8).
