# Supplementary material for: Rapid Freezing Enables Aminoglycosides To Eradicate Bacterial Persisters via Enhancing Mechanosensitive Channel MscL-Mediated Antibiotic Uptake
Source: mBio. 2020 Feb 11;11(1):e03239-19. doi: 10.1128/mBio.03239-19 (PMC7018644; doi:10.1128/mBio.03239-19)
Supplement: FIG S7 [file mBio.03239-19-sf007.pdf]

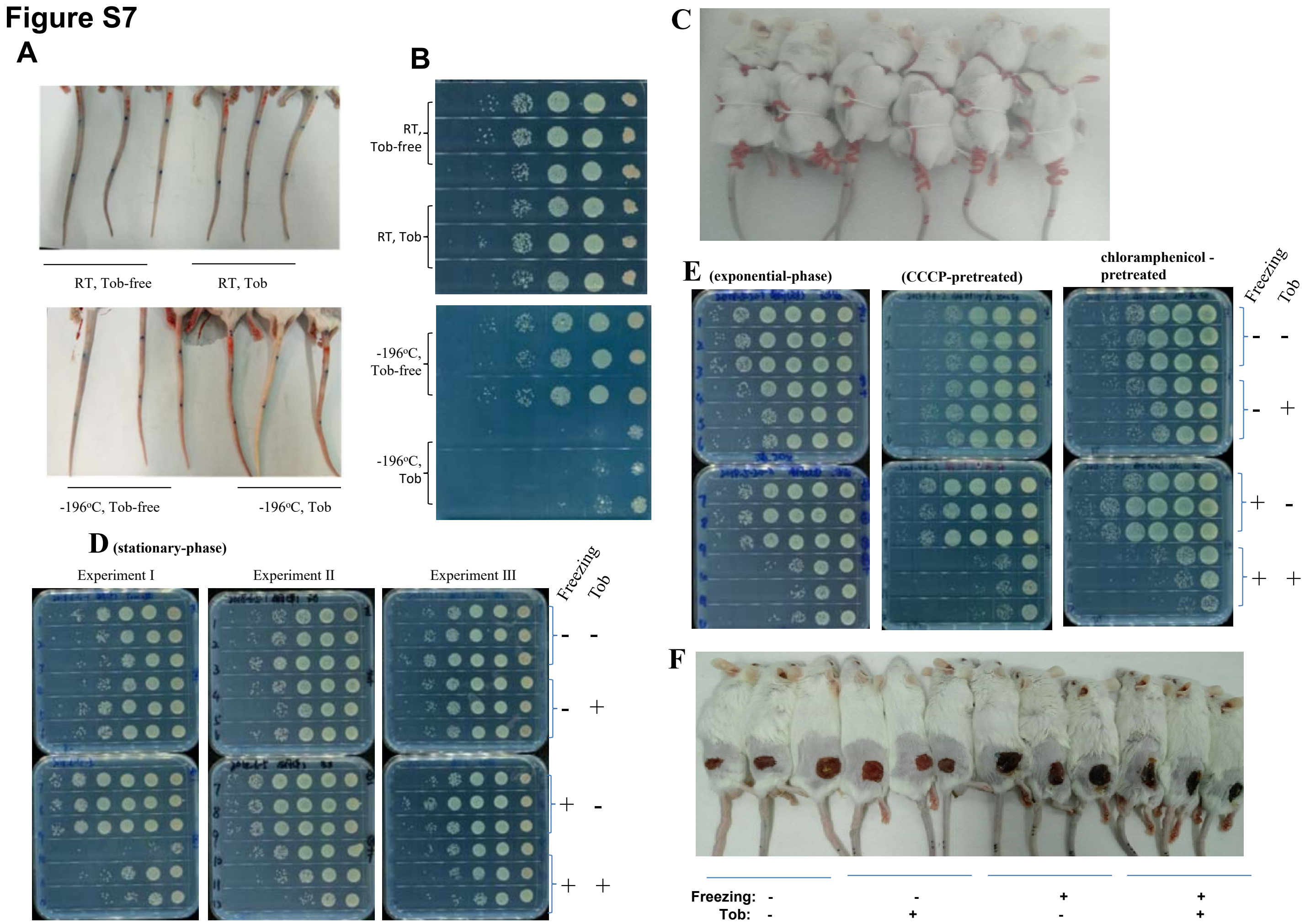

Fig. S7 Freezing facilitates tobramycin to kill *P. aeruginosa* in mouse model

(**A**) Mouse tails after the indicated treatment. Note that the frozen tails appear slightly red in color. (**B**) Survival of stationary-phase *P. aeruginosa* cells on LB agar dishes after the cells were mixed with 100 μg/mL tobramycin, plated on the tail of mice, and subjected to freezing in liquid nitrogen for 2 min. Tails were then cut and homogenized, with the lysates being spot-plated onto LB agar dishes. Representative results from three independent experiments are shown here. Quantification results are shown in **Fig. 5B**. (**C**) Photo of mice with a bandage on their acute skin wounds. (**D**) Survival of stationary-phase *P. aeruginosa* cells on LB agar dishes after the cells were treated as following: stationary-phase *P.aeruginosa* cells were seeded on the wound of anesthetized mice, and tobramycin-containing medium were added and fully absorbed before subjected to freezing on the wound; afterwards, wound was bandaged (refer to Panel C), and mice were housed over-night. Three independent experimental results are shown here. (**E**) Survival of exponential-phase *P. aeruginosa* cells collected from the mouse acute skin wound model experiments. Cells were pre-treated without (left part) or with CCCP (middle part) or chloramphenicol (right part), plated on the skin wound of mice and then subjected to the indicated treatments. After mice were housed over-night, the whole scab on the wound site was carefully removed and homogenized, and subjected to bacterial survival assay. Representative results from three independent experiments are shown here. (**F**) Mice of an acute skin wound model. After bacterial seeding and treatment, wounds were bandaged (refer to panel C) and mice were housed over-night before performing bacterial survival assay (for detail, refer to the Methods section).
