# Supplementary material for: Rapid Freezing Enables Aminoglycosides To Eradicate Bacterial Persisters via Enhancing Mechanosensitive Channel MscL-Mediated Antibiotic Uptake
Source: mBio. 2020 Feb 11;11(1):e03239-19. doi: 10.1128/mBio.03239-19 (PMC7018644; doi:10.1128/mBio.03239-19)
Supplement: FIG S8 [file mBio.03239-19-sf008.pdf]

Figure S8

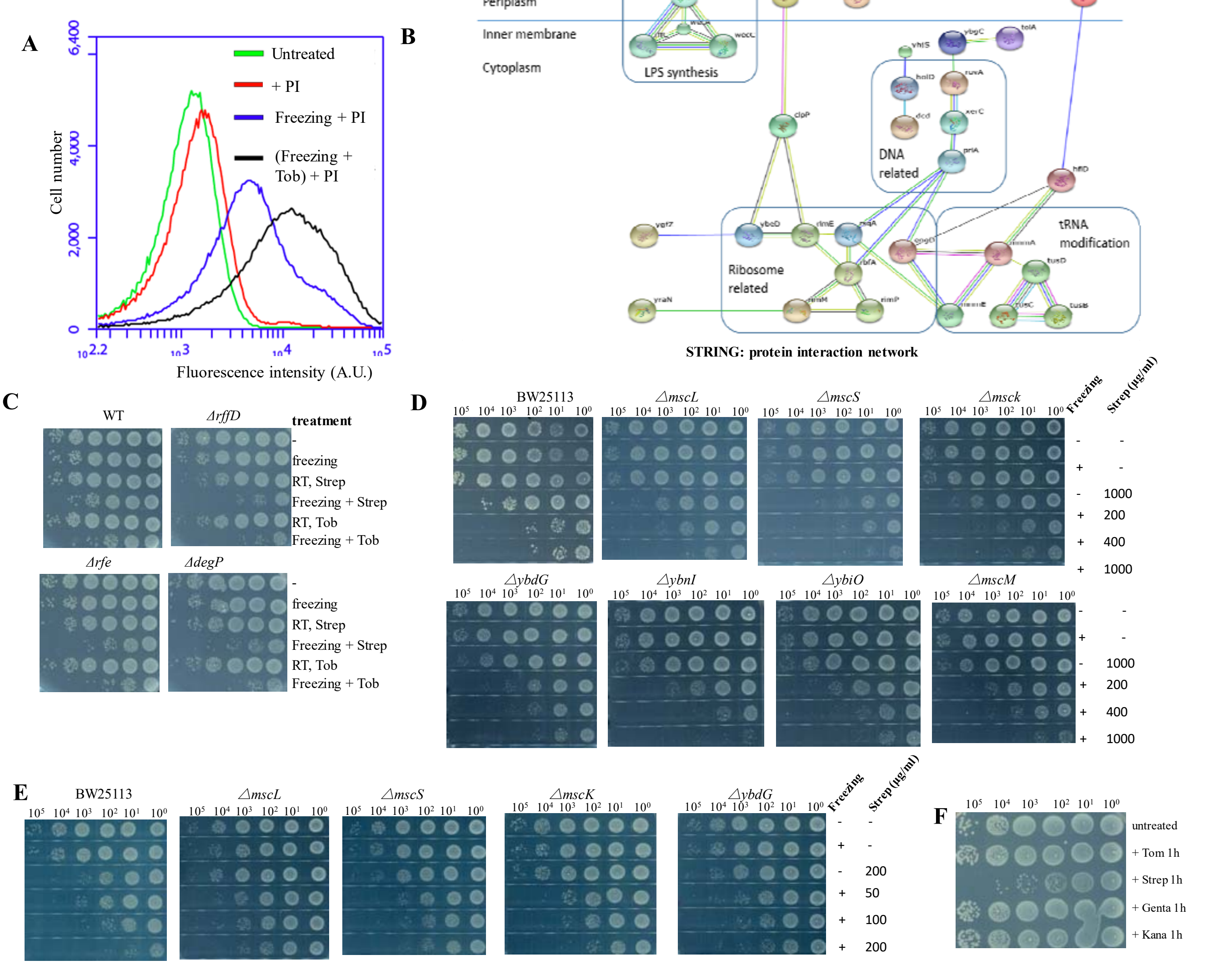

Fig. S8 Freezing-induced *E. coli* cell membrane damage and implications for the involvement of *mscL* gene in freezing-induced aminoglycoside potentiation.

(**A**) Flow cytometry analysis of exponential-phase *E. coli* cells, which were frozen in the absence or presence of tobramycin (25 µg/mL), thawed and incubated with propidium iodide (PI, 50 µg/mL) for 10 min before sorting analysis. (**B**) Functional classification of candidate genes by STRING, suggesting that

freezing sensitivity-related genes may be linked to tRNA modification, ribosome biogenesis, DNA, membrane biogenesis and protein quality control. The Keio collection for *E. coli* single-gene deletion library was subjected to freezing treatment, with those freezing-sensitive mutants being identified. Candidate genes were subjected to bioinformatic analysis for functional annotation and classification using such on-line tools (KEGG, http://www.genome.jp/kegg/; STRING, http://string-db.org; UNIPROT, http://www.uniprot.org/). (**C**) Survival of exponential-phase *E. coli* cells of indicated genotypes. Wild type (BW25113) and single-gene deletion strains of *E. coli* were cultured to an OD_{600} of 0.5 and then subjected to the freezing treatment in the absence or presence of tobramycin before cell survival assay. (**D**) Survival of stationary-phase *E. coli* cells of indicated genotypes after the cells were subjected to three cycles of freezing treatment in liquid nitrogen for 10 sec in the presence of streptomycin at indicated concentrations. (**E**) Survival of exponential-phase *E. coli* cells of indicated genotypes after the cells were subjected to freezing treatment in liquid nitrogen for 10 sec in the presence of streptomycin at indicated concentrations. (**F**) Antibiotic sensitivity test of MJF612 strain to aminoglycosides. The exponential-phase MJF612 cells were agitated for 1 h in the presence of indicated antibiotics and then subjected to cell survival assay. MJF612 (Frag1 *ΔmscL*::cm, *ΔmscS*, *ΔmscK*::kan, *ΔybdG*:: aprD) encodes a kanamycin-resistance gene, which is apparently cross-resistant to tobramycin and gentamicin as shown here.
