# Supplementary material for: Rapid Freezing Enables Aminoglycosides To Eradicate Bacterial Persisters via Enhancing Mechanosensitive Channel MscL-Mediated Antibiotic Uptake
Source: mBio. 2020 Feb 11;11(1):e03239-19. doi: 10.1128/mBio.03239-19 (PMC7018644; doi:10.1128/mBio.03239-19)
Supplement: FIG S9 [file mBio.03239-19-sf009.pdf]

Figure S9

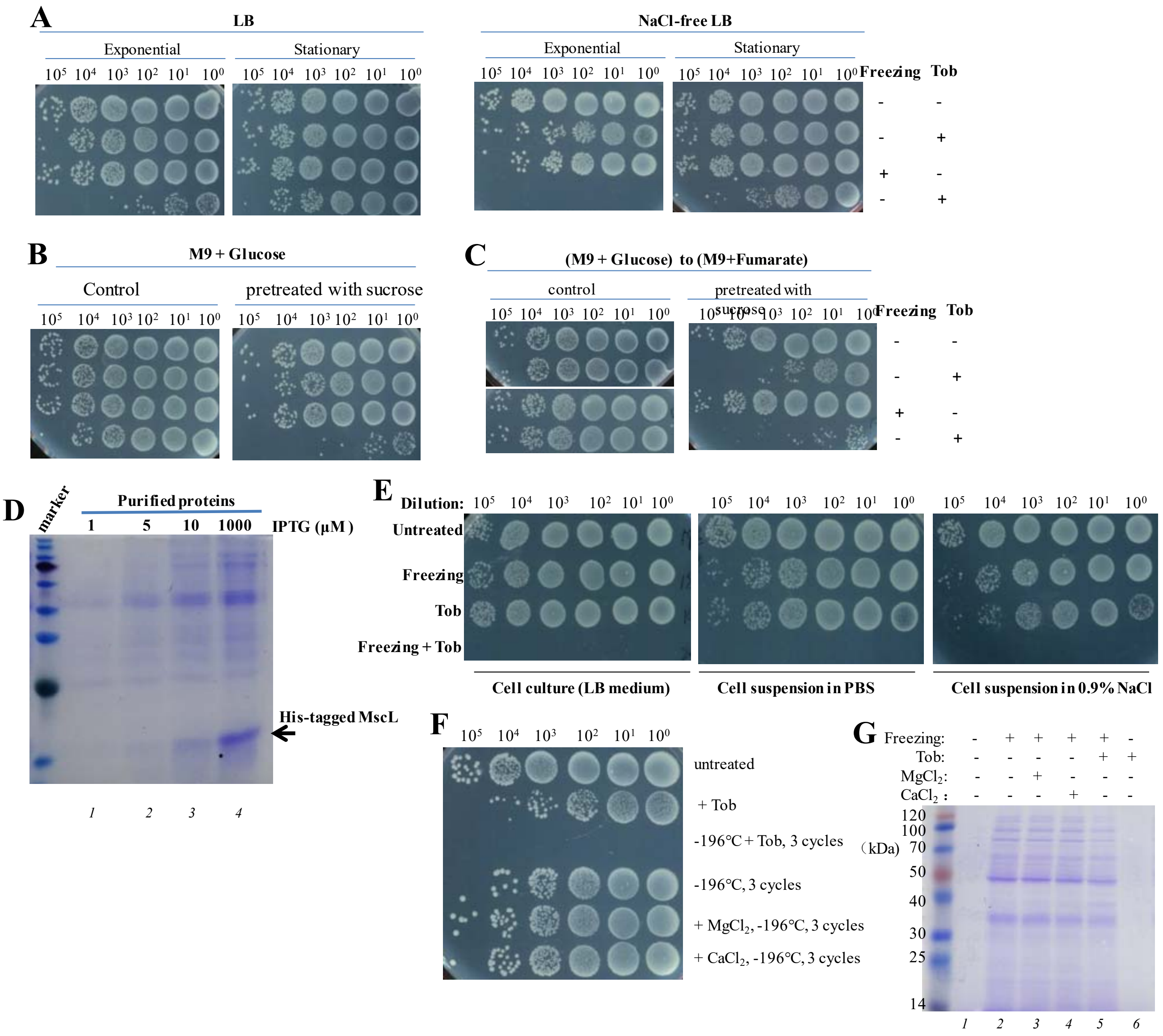

Fig. S9 MscL channel mediates the uptake of streptomycin in *E. coli* cells upon freezing and such uptake is inhibited by Ca^{2+}/Mg^{2+}

(**A**) Survival of *E. coli* BW25113 cells following two cycles of 30 s treatment of tobramycin (25 μg/ml) and freezing (in ethanol prechilled at -80°C). Cells were cultured in LB (left part) or NaCl-free LB medium (right part) to both exponential- and stationary-phase stages and then adjusted to an OD_{600} of 0.6 before subjected to the same combined treatment. (**B**, **C**) Survival of *E. coli* BW25113 cells following the same treatment as described in Panel A. Cells were cultured in M9 + 5 g/L glucose to an OD_{600} of 0.6, with or without transferring to M9 + 2 g/L fumarate for 4 h (panel C), and then subjected to osmotic shock treatment with 1.25 M sucrose for 10 min before the combined treatment of tobramycin and freezing. (**D**) SDS-PAGE analysis of MscL protein that was purified from MJF612 cells complementarily expressing MscL upon induction with elevated concentrations IPTG. Protein purification was performed by Ni-NTA agarose chromatography. (**E**) Survival of stationary-phase *E. coli* cells on LB agar dishes after the cells were mixed with 100 μg/mL tobramycin in different suspensions (LB, PBS or 0.9% NaCl) and subjected to three cycles of freezing treatment in liquid nitrogen for 10 sec. Note: PBS buffer contains 45 mM K^+ and 150 mM Na^+. (**F**) Survival of *E. coli* BW25113 exponential-phase cells ($OD_{600} \approx 0.6$) following three cycles of 10-sec freezing treatment in the presence of tobramycin, 10 mM Ca^{2+} or Mg^{2+}. (**G**) SDS-PAGE analysis results of the supernatant of exponential-phase *E. coli* cells after experiencing the same treatment as described in Panel F. Proteins were precipitate with 10% trichloroacetic acid before electrophoresis.
